# Mapping of Language-and-Memory Networks in Patients With Temporal Lobe Epilepsy by Using the GE2REC Protocol

**DOI:** 10.3389/fnhum.2021.752138

**Published:** 2022-01-06

**Authors:** Sonja Banjac, Elise Roger, Emilie Cousin, Chrystèle Mosca, Lorella Minotti, Alexandre Krainik, Philippe Kahane, Monica Baciu

**Affiliations:** ^1^Université Grenoble Alpes, CNRS LPNC UMR 5105, Grenoble, France; ^2^Université Grenoble Alpes, UMS IRMaGe CHU Grenoble, Grenoble, France; ^3^Université Grenoble Alpes, Grenoble Institute of Neuroscience ‘Synchronisation et modulation des réseaux neuronaux dans l’épilepsie’ & Neurology Department, Grenoble, France

**Keywords:** language, memory, temporal lobe epilepsy, fMRI mapping, preoperative assessment, surgery

## Abstract

Preoperative mapping of language and declarative memory functions in temporal lobe epilepsy (TLE) patients is essential since they frequently encounter deterioration of these functions and show variable degrees of cerebral reorganization. Due to growing evidence on language and declarative memory interdependence at a neural and neuropsychological level, we propose the GE2REC protocol for interactive language-and-memory network (LMN) mapping. GE2REC consists of three inter-related tasks, sentence generation with implicit encoding (GE) and two recollection (2REC) memory tasks: recognition and recall. This protocol has previously been validated in healthy participants, and in this study, we showed that it also maps the LMN in the left TLE (*N* = 18). Compared to healthy controls (*N* = 19), left TLE (LTLE) showed widespread inter- and intra-hemispheric reorganization of the LMN through reduced activity of regions engaged in the integration and the coordination of this meta-network. We also illustrated how this protocol could be implemented in clinical practice individually by presenting two case studies of LTLE patients who underwent efficient surgery and became seizure-free but showed different cognitive outcomes. This protocol can be advantageous for clinical practice because it (a) is short and easy to perform; (b) allows brain mapping of essential cognitive functions, even at an individual level; (c) engages language-and-memory interaction allowing to evaluate the integrative processes within the LMN; (d) provides a more comprehensive assessment by including both verbal and visual modalities, as well as various language and memory processes. Based on the available postsurgical data, we presented preliminary results obtained with this protocol in LTLE patients that could potentially inform the clinical practice. This implies the necessity to further validate the potential of GE2REC for neurosurgical planning, along with two directions, guiding resection and describing LMN neuroplasticity at an individual level.

## Highlights

- GE2REC robustly maps LMN network in TLE patients.
- GE2REC allows assessing LMN at an individual level.
- GE2REC assess several memory processes.
- Compared to controls, left TLE show reduced LMN integration.

## Introduction

Studies on temporal lobe epilepsy (TLE), the most common form of epilepsy, cite problems with episodic memory and naming or verbal fluency among the most frequent concomitant cognitive deficits (Bell et al., 2011; Bartha-Doering and Trinka, 2014; Zhao et al., 2014; Jaimes-Bautista et al., 2015; Tramoni-Negre et al., 2017; Dutta et al., 2018). The additional risk of the cognitive deficit is imposed by surgery that almost 30% of TLE patients need due to drug-resistant seizures (Sherman et al., 2011; Borger et al., 2021). Even though standard surgical procedures (such as anterior temporal lobectomy) have shown to be generally efficient in terms of seizure freedom (McIntosh, 2004; Téllez-Zenteno et al., 2005; Aull-Watschinger et al., 2008; Mohan et al., 2018), TLE patients can also “pay a considerable cognitive price” postoperatively, that can have an impact on their life-quality (Helmstaedter, 2004, 2013; Baxendale and Thompson, 2018). Specifically, a meta-analysis reported that 44% of left TLE (LTLE) patients faced verbal memory and 34% naming decline (Sherman et al., 2011). Hence, the main goal of preoperative assessment is to perform a cost-benefit analysis and evaluate the risks of cognitive decline versus the potential seizure freedom (Massot-Tarrús et al., 2019). In addition to clinical and neuropsychological characteristics of patients that are essential in the presurgical assessment (Baxendale et al., 2013; Jarčušková et al., 2020), functional MRI (fMRI) can predict postoperative cognitive performance and decline, and it is frequently used for preoperative assessment (Binder et al., 2010; Bonelli et al., 2010; Sidhu et al., 2015; Strandberg et al., 2017; You et al., 2019). It should be noted that this paper focuses on fMRI application in determining brain regions that are active during a specific task. Nevertheless, other mapping methods using fMRI can also be used in the presurgical evaluation. For instance, studies show clinical application of functional connectivity (Bettus et al., 2010; Pravatà et al., 2014; Tracy and Doucet, 2015; DeSalvo et al., 2020; Foesleitner et al., 2021) and effective connectivity (Vaudano et al., 2021).

The preoperative mapping of cognitive functions under risk is even more necessary because TLE patients show variable probability and degree of cerebral reorganization of language and memory networks at inter- and intra-hemispheric level (Rosenberger et al., 2009; Sidhu et al., 2013; Baciu and Perrone-Bertolotti, 2015; Chang et al., 2017; Neudorf et al., 2020) which is also sometimes followed by atypical cognitive profiles (Cabrera et al., 2018).

Given the frequent episodic memory and naming difficulties TLE patients are facing, standard preoperative task-based functional magnetic resonance imaging (fMRI) protocols for these patients mainly focused on either (episodic or verbal) memory (Cheung et al., 2009; Bonelli et al., 2010; Dupont et al., 2010; Limotai et al., 2018) or language processes (Binder et al., 2011; Benjamin et al., 2017; Trimmel et al., 2019). Although these studies bring promising results in predicting a postoperative decline, they do not address the question of cognitive functioning holistically. Namely, everyday life functioning is not based on isolated functions, as they are often studied, but rather on the cognitive synergy. Indeed, the interaction between functions has already been acknowledged and demonstrated (Kellermann et al., 2016; Tosi et al., 2020), especially the necessity of language to rely on the declarative memory (Clark and Marshall, 1981; Larsen et al., 2002; Stalnaker, 2002; Park et al., 2011; Duff and Brown-Schmidt, 2012). Moreover, the importance of interactions between language and memory performance in TLE lateralization has also been shown (Roger et al., 2020b). Hence, it is vital to consider the integrality of patients’ performance (Tosi et al., 2020). TLE patients are a good model for exploring cognitive function interaction because their epileptogenic zone is situated in the temporal lobe, which plays an essential role in both language and memory (Tracy and Boswell, 2008; Drane and Pedersen, 2019). Furthermore, TLE is often followed by hippocampal atrophy (Thom and Bertram, 2012; Barr, 2015). Although the role of hippocampal structure in memory processes is widely acknowledged (Moscovitch et al., 2006, 2016; Ranganath and Ritchey, 2012), there is growing evidence of its implication in the language (Whitney et al., 2009; Kurczek et al., 2013; Moscovitch et al., 2016; Piai et al., 2016), and it is even proposed to be the interface between language and memory (Duff and Brown-Schmidt, 2012), or a modulator between language and default mode networks (Nenning et al., 2021).

To synthesize, the main concern of the presurgical investigation in TLE patients is the postsurgical quality of everyday functioning based on this cognitive synergy, rather than just one function. Therefore, there is a need to design a presurgical protocol to address this cognitive interaction and not just map two functions separately. Although many protocols that map both language and memory exist and have clear advantages (Deblaere et al., 2002; Aldenkamp et al., 2003; Buck et al., 2020), they examine these functions separately, trying to segregate them rather than acknowledging their entanglement.

Apart from eliciting interaction between language and declarative memory, a presurgical fMRI protocol would have to allow to map the structures that are of importance for these functions, but also those that are of importance for TLE patients – temporal lobe and hippocampus (Binder et al., 2011; Benjamin et al., 2017; Buck and Sidhu, 2020). For this diagnostic purpose, an fMRI protocol should provide information regarding the dominant hemisphere and localization of the language-and-memory network (LMN) in relation to the potential epileptogenic zone (Ghosh et al., 2010). In doing so, it should be acknowledged that language and declarative memory interaction is based on a widely distributed network (Roger et al., 2020a). A presurgical protocol is additionally required to be applicable in the clinical setting that is quite different from the experimental environment. A protocol appropriate for a clinical setting should be short, easy to perform, and easy to analyze (Cabrera et al., 2018). Moreover, in presurgical evaluation, the level of interest is an individual patient, and studies on fMRI protocols usually report only group-level results (Deblaere et al., 2002; Aldenkamp et al., 2003; Buck et al., 2020; except Binder et al., 2011; Benjamin et al., 2017). However, it was shown that group-level effects are not always relevant or valid on the individual subject level (Seghier and Price, 2016). Therefore, a presurgical protocol needs to demonstrate the ability to activate the main network and critical regions on an individual level. This is especially important for structures, such as the hippocampus, susceptible to geometric distortions, signal loss, and low signal-to-noise ratio (Powell and Duncan, 2005; Haag and Bonelli, 2013; Buck et al., 2020). Finally, the results of an fMRI protocol should be integrated within a wider set of presurgical evaluations, above all neuropsychological scores, to interpret and comprehend a complex clinical image of a patient.

In the present study, we assessed the utility of the GE2REC fMRI protocol for mapping a LMN as a cerebral substrate of interaction between language and declarative memory in LTLE. The main objective is twofold: (a) to determine if this protocol is sensitive to differences between LTLE patients and healthy controls (HC), thus investigating neuroplasticity from a more fundamental and theoretical perspective; and (b) to provide some initial paths, currently only preliminary, of the potential benefit while adding the LMN information at an individual level during the neurosurgical planning (resection guidance) and of the cognitive mapping in this context. Finally, we will present two case studies to illustrate the application of the GE2REC protocol at an individual level before and after surgery. This also allowed us to explore the effectiveness of functional-anatomical reorganization.

## Materials and Methods

### Participants

The present study included eighteen LTLE patients, candidates for curative surgery (age 35 ± 10.9; 10 females; 17 right-handed), and nineteen healthy controls (HC, age 21.2 ± 2.97; 8 females; all right-handed) without neurological and psychiatric deficits. Patients were diagnosed with drug-resistant LTLE by neurologists based on a synthesis of several evaluations (clinical, scalp/depth-EEG, MRI/PET scan) following the recommendations of the ILAE (International League Against Epilepsy) committee report (Wieser et al., 2001). The Edinburgh Handedness Inventory (Oldfield, 1971) was used to determine handedness in LTLE. Ten patients subsequently underwent left anterior temporal lobectomy. Postoperative anatomical data was available for seven of those patients. All participants were French native speakers with normal or corrected-to-normal vision. One HC participant was excluded from the fMRI analyses due to the high number of artifacts in the data. This clinical experimentation was carried out under the French law (Jardé, Décret n°2016-1537 16/11/2016 from 17/11/2016). The Ethics committee approved the project for the protection of persons (CPP 09-CHUG-14; MS-14-102). All participants gave their written informed consent to participate in the study. HC received financial compensation for their participation. For patients, fMRI evaluations were part of their presurgical assessment. Demographic, clinical, and neuropsychological details of all LTLE patients are presented in Table 1.

**TABLE 1:** Demographic, clinical, and neuropsychological data for LTLE patients.

	Demographic information			Clinical data					Language and memory scores							Postsurgical data			
			
	Sex	Age	Hd.	HA	ASO	ED	AED	SF	VCI	DO 80	SFL	AMI	VMI	IMI	DMI	NTD	RCI	EC	rLMN%
P1	M	28	R	Yes	1	27	2	M	0.28	0.70	–0.24	0.67	1.56	1.08	1.34	1	–0.16	Ia	3.98
P2	M	45	R	Yes	40	5	4	D	–0.28	–1.30	–0.67	–0.52	0	–0.08	–0.28	2	–1.20	Ia	5.25
P3	M	24	R	Yes	17	7	3	W	/	–1.30	–0.34	**/**	/	/	/	/	/	/	/
P4	M	24	L	Yes	16	8	3	M	–0.41	–**2.20**	–**2.17**	–**3.47**	–0.61	–**1.88**	–**2.33**	/	/	/	/
P5	M	27	R	No	20	7	3	D	0.00	–0.03	–0.34	1.34	–1.13	0.13	0.00	/	/	/	/
P6	F	26	R	Yes	13	13	3	M	–0.52	–**2.05**	–1.89	0.28	0	–0.47	0.47	3	–3.21	Ia	6.18
P7	F	43	R	No	12	31	3	M	0.28	–1.30	–1.46	0.09	–**1.75**	–0.08	–1.13	4	–4.36	IVc	23.46
P8	F	43	R	Yes	3	40	1	M	0.52	–0.3	–**2.05**	–0.28	–0.2	–0.47	–0.28	0	1.84	Id	8.24
P9	F	38	R	Yes	10	28	3	M	–1.56	–**5.30**	–0.98	0.47	0	0.41	0.08	3	–3.42	Ia	11.81
P10	M	24	R	Yes	20	4	4	>M	–0.13	–**4.30**	1.66	0.61	–0.2	0.47	–0.08	3	–0.27	Ia	/
P11	F	54	R	No	52	2	2	W	0.00	0.67	1.39	0.33	–1.08	–0.99	–0.28	2	–6.53	IVc	/
P12	F	37	R	No	35	2	2	>M	0.00	–0.30	0.34	1.34	–**2.05**	–0.47	–0.74	/	/	/	/
P13	M	24	R	No	23	1	2	M	0.52	–1.3	0.64	0.08	–0.61	–0.28	–0.47	1	1.43	Ia	/
P14	F	32	R	Yes	29	3	2	D	–1.08	–1.46	–0.98	–1.08	–1.18	–1.56	–1.41	1	–1.02	Ia	8.85
P15	F	23	R	Yes	8	15	2	W	0.81	0.26	–0.34	**2.05**	0.81	1.75	1.48	/	/	/	/
P16	M	58	R	No	14	44	2	M	1.08	–1.3	–1.28	0.33	0.47	0.47	0.47	/	/	/	/
P17	F	37	R	Yes	36	1	5	D	–0.28	–0.17	–1.46	–0.99	–1.6	–1.48	–**1.65**	/	/	/	/
P18	F	41	R	Yes	15	26	1	D	/	/	/	/	/	/	/	/	/	/	/
Mean (SD)	10F/8M	34.9 (10.9)	17R/1L	12Y/6N	20.2 (13.5)	14.6 (14.2)	2.6 (1)	5D/3W/8M/2 < M	–0.05 (0.7)	–1.25 (1.62)	–0.6 (1.1)	0.08 (1.3)	–0.47 (1)	–0.21 (1)	–0.30 (1)	2 (1.2)	–1.69 (2.6)	7 Ia/1 Id/2 IVc	9.68 (6.6)
GD Mean (SD)	4F/2M	38.3 (11.6)	6R/0L	4Y/2N	24.5 (17.4)	13.8 (12.7)	3.3 (1)	1D/1W/3M/1 > M	–0.4 (0.6)	–2.3 (2.2)	–0.3 (1.5)	0.2 (0.4)	–0.5 (0.7)	–0.1 (0.5)	–0.2 (0.5)	2.8 (0.8)	–3.2 (2.2)	4 Ia/2 IVc	11.67 (8.4)
RD Mean (SD)	2F/2M	31.8 (8.2)	4R/0L	3Y/1N	14 (14.1)	17.8 (19)	2.3 (1.3)	1D/3M	0.1 (0.8)	–0.6 (1)	–0.7 (1.1)	–0.2 (0.7)	–0.1 (1.2)	–0.3 (1.1)	–0.2 (1.1)	0.8 (0.5)	0.5 (1.3)	3 Ia/1 Id	7.02 (2.6)

*Postsurgical data is presented for patients that underwent surgery and based on these data, they were divided into groups with greater and reduced postsurgical cognitive decline.*

*F, female; M, male; Age, age at the time of examination; Hd., handedness evaluated with Edinburgh quotient (Oldfield, 1971); L, left, R, right; HA, hippocampal atrophy; ASO, age of onset of seizures; ED, epilepsy duration; AED, number of epileptic drugs taken; SF, seizure frequency: D, seizures occurring on a daily basis; W, seizures occurring on a week basis; M, seizures occurring on a monthly basis; >M, seizures occurring once in couple of months; VCI, standardized score of verbal comprehension index (Wechsler, 2008); DO80, standardized score for French version of naming task (Deloche and Hannequin, 1997); SFL, semantic fluency, z core of performance on the task of categorical word generation (Godefroy and GREFEX, 2008); AMI, standardized score of auditory memory (Wechsler, 2009); VMI, standardized score of visual memory (Wechsler, 2009); IMI, standardized score of immediate memory (Wechsler, 2009); DMI, standardized score for delayed memory (Wechsler, 2009); NTD, number of tests with significant postsurgical decline; RCI, average reliable change index; EC, Engel class (Engel, 2014); Ia, completely seizure-free; Id, generalized convulsions with antiseizure drug discontinuation only; IVc, seizures got worse; rLMN%, the percentage of preoperative LMN that was resected; GD, greater postsurgical cognitive decline; RD, reduced postsurgical cognitive decline.*

*Scores marked in bold were pathological (p ≤ 0.05).*

#### Case Studies Patients

To illustrate the potential of the GE2REC protocol at an individual level in clinical practice, we present two case studies of LTLE patients who underwent left anterior temporal lobectomy and had different ages of seizures onset, epilepsy duration, seizure frequency, and the number of antiepileptic drugs. Hereafter, we present a detailed description of P1 and P2.

Patient 1 (P1) is a 28-year-old man with left mesio-temporal epilepsy starting at 9 months (early onset of seizures). The patient reported two types of seizures. A signal pain symptom characterized the first type without contact break or aphasia, which occurred several times per month. The second, a less common type of seizure, started with the same sensation but was followed by contact break, dystonic manifestations of the upper limbs, rubefaction, and chewing automatism lasting about a minute. This type of seizure was followed by a post-critical period with word retrieval difficulties and fatigue. MRI revealed atrophy of the left hippocampus and ipsilateral temporal pole with blurring phenomenon (Naves et al., 2015).

Patient 2 (P2) was a 45-year-old male with left mesio-temporal epilepsy starting at 40 years of age (late onset of seizures). Seizures were characterized by contact break and distressing feeling of heat, followed by fixed gaze, chewing automatisms, incoherent speech, and word retrieval difficulties. Patient also reported aura as epigastric sensation, *déjà vu* and *déjà vécu* phenomena. Initially, seizures occurred several times per week, but as the illness progressed, the frequency increased, with seizures occurring several times per day. Presurgical MRI showed hippocampal atrophy, polar and medial temporal hypometabolism, predominantly to the left.

For both patients, combinations of various antiepileptic drugs were initially administrated without significant benefice on stopping seizures. Therefore, they were considered pharmaco-resistant and were proposed a surgery. Functional MRI, neuropsychological and language investigations were a part of their preoperative and postoperative assessment. Demographic, clinical, neuropsychological, and functional activation of patients before and after surgery are presented in Table 2.

**TABLE 2:** Demographic, clinical, neuropsychological, language and functional MRI data in P1 and P2 before and after surgery.

	P1			P2		
**Demographic information**						
Gender	Male			Male		
Age	28			45		
Education	Vocational high-school			Vocational high-school		
Profession	Butcher			Carpenter		
**Epilepsy medical history**						
Type of epilepsy	Mesial LTLE			Mesial LTLE		
Hippocampal atrophy	Yes			yes		
Age of epilepsy onset	9 months			40 years		
Epilepsy duration	27			5		
Frequency of seizures	Monthly			Daily		
Number of AEDs	2			4		
**Postoperative seizure outcome**						
Engel class	Ia completely seizure-free			Ia completely seizure-free		
**Neuropsychological assessment**	**Before surgery**	**After surgery**		**Before surgery**	**After surgery**	
VCI	0.28	0.28	=	–0.28	–0.41	↓
DO-80	0.7	–1.3	↓*	–1.3	**–2.3**	↓*
SFL	–0.24	–0.62	↓	–0.67	–0.98	↓
AMI	0.67	0.88	↑	–0.52	–1.08	↓
VMI	1.56	1.88	↑	0	–0.08	=
IMI	1.08	1.34	↑	–0.08	–0.74	↓*
DMI	1.34	1.65	↑	–0.28	–0.61	↓
TMT B-A	–0.57	–0.28	↑	**–2.14**	–1.38	↑
Handedness	R (+100%)			R (+20%)		
**RECO responses**						
Correct	75%	77.5%	↑	80%	72.5%	↓
Incorrect	21.3%	12.5%	↓	16.3%	20%	↑
**fMRI – LI**						
GE frontal lobe	0.71	0.84	←	0.71	0.29	→
GE temporal lobe	0.66	0.94	←	0.53	0.33	→
GE hippocampus	0.73	/		0.38	/	
RECO frontal lobe	–0.086*	0.67	←	0.081*	0.55	←
RECO temporal lobe	0.41	0.51	←	0.003	**–0.43**	→
RECO hippocampus	**–0.39**	/		**–0.63**	/	
RA frontal lobe	0.59	0.93	←	0.7	**–0.24** *	→
RA temporal lobe	0.64	0.83	←	0.46	**–0.45** *	→
RA hippocampus	**–0.38**	/		0.69	/	

Arrows for neuropsychological and language assessment, recognition performance and LIs indicate the direction of change (increase, decrease, shift to left or right) after surgery. Neuropsychological and language changes marked with * indicate a significant score change (using 90% confidence interval). Scores marked in bold were pathological. LIs marked with * indicate significantly different values (p < 0.05) compared to healthy controls, based on modified Crawford test (Crawford and Garthwaite, 2002). LI values in bold indicate right hemispheric predominance (Seghier, 2019).

*LTLE, left temporal lobe epilepsy; AED, number of epileptic drugs taken; VCI, standardized score of verbal comprehension index (Wechsler, 2008); DO80, standardized score for French version of naming task (Deloche and Hannequin, 1997); SFL, semantic fluency, z core of performance on the task of categorical word generation (Godefroy and GREFEX, 2008); AMI, standardized score of auditory memory (Wechsler, 2009); VMI, standardized score of visual memory (Wechsler, 2009); IMI, standardized score of immediate memory (Wechsler, 2009); DMI, standardized score for delayed memory (Wechsler, 2009); TMT B-A, standardized score of Trail Making Test B-A (Godefroy and GREFEX, 2008); LI, Lateralization index; GE, sentence generation with implicit encoding; RECO, recognition of items; RA, recall.*

### Neuropsychological and Language Assessment in Patients

Left TLE patients underwent neuropsychological, and language assessments carried out by a neuropsychologist and a speech therapist. The present study used the assessment results to test the cognitive efficiency of obtained cerebral activated networks. The following cognitive scores were used in the analyses: (a) general cognitive level (IQ) composed of: verbal comprehension index (VCI) (WAIS IV, Wechsler, 2008); (b) language scores: naming (DO80; Deloche and Hannequin, 1997), semantic fluency (SFL; Godefroy and GREFEX, 2008); (c) memory scores: auditory (AMI) and visual memory indices (VMI; WMS IV, Wechsler, 2009), immediate (IMI) and delayed memory indices (DMI; WMS IV, Wechsler, 2009) and only for case studies (d) executive functioning: Trail Making Test B-A (Godefroy and GREFEX, 2008). Test scores were standardized by gender, age, and sociocultural level based on validation data of each neuropsychological test used. Standardized scores were considered as pathological if they were equal or lower than –1.65 SD, corresponding to a threshold of *p* ≤ 0.05 (Roger et al., 2019). Detailed data on patients’ cognitive performance is presented in Tables 1, 2. For patients who underwent surgery and had postoperative neuropsychological evaluation, we calculated reliable change indices at a 90% confidence interval (Baxendale and Thompson, 2005; Morley, 2017). To compare patients in our sample experiencing greater and reduced postoperative cognitive decline, we separated the patients for whom the data was available into two groups. We focused on the language (DO80 and SFL) and memory scores (AMI, IMI, DMI, IMI). Patients were considered to show greater postoperative cognitive decline if two or more of these scores were significantly reduced after surgery. Those with the decline in less than two tests were considered as showing reduced (or no) cognitive decline. Table 1 presents the clinical characteristics of these patients.

### Functional MRI Assessment of Language and Memory

**FIGURE 1:**
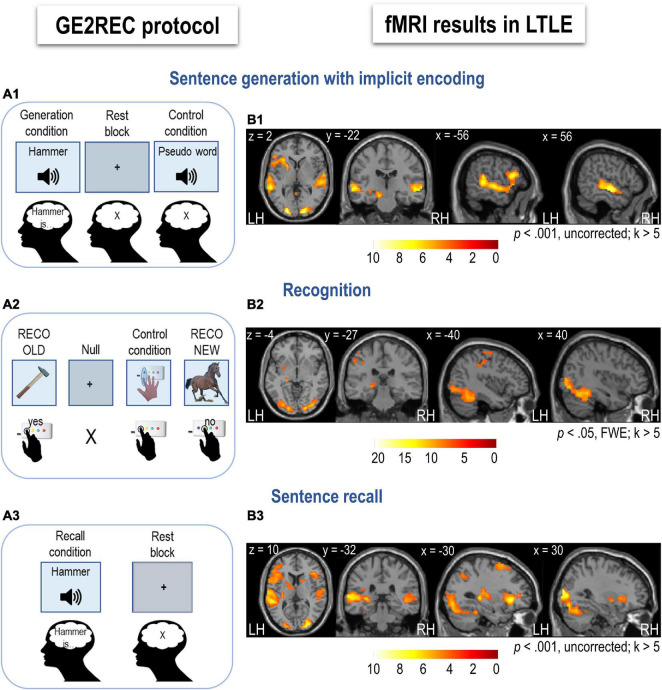
Schematic illustration of the GE2REC protocol and activation maps for each task in left temporal lobe epilepsy (LTLE) patients (*N* = 18). **(A1)** Sentence generation with implicit encoding with a block design. Items were presented in the auditory modality during Task (words) and Control (pseudoword) and in the visual modality during Rest (central cross). Participants were required to covertly generate sentences during Task, listen to the pseudoword during Control, and fixate the cross during Rest. **(A2)** Recognition task with event-related design. Items were presented in visual modality during Task (pictures), Control (instruction image), and Null events (central cross). Participants were required to respond whether they recognized an object in a given picture as a word presented in the previous run (change of modality from auditory to visual). They responded by pressing two box buttons (yes, no). During Control, they were asked to press the button, as shown in the picture. **(A3)** Recall task with block design. Items were presented in the auditory modality during Task (words) and in the visual modality during Rest (central cross). Participants were required to recall and covertly repeat the sentences they generated in the first run in response to presented words. Participants were asked to fixate the cross during Rest. The activation maps obtained during sentence generation **(B1)**, recognition **(B2)**, and recall **(B3)** in LTLE patients are projected onto a 2D template using xjview toolbox (https://www.alivelearn.net/xjview). The color scale indicates the *T* value. For illustration, LMN is presented at a more permissive threshold for sentence generation and recall (*p* < 0.001, *k* > 5). LH, left hemisphere; RH, right hemisphere.

The experimental protocol was developed using E-prime software (Psychology Software Tools, Pittsburgh, PA, United States). Participants were given a general description of the procedure before entering the fMRI. To ensure that participants performed implicit encoding and did not use individual strategies for memorizing words, they were only provided with the description of the sentence generation task. They were told that this task would be followed by two more, and they were given the information regarding the modality of the tasks and the manner of responding (i.e., using the response box). Participants remained unaware of the actual content of the memory task and that tasks are connected. A schematic illustration of all tasks is presented in Figure 1A. The description of the protocol is provided below. For a more detailed explanation, please see Banjac et al. (2020).

#### Sentence Generation With Implicit Encoding Stimuli and Task (GE)

In the first run, the participants heard words through a headset. Upon hearing a word, they were asked to covertly generate sentences related to the word they had just heard. They had to produce the sentences related to the heard word until the next word was heard. The words-stimuli were auditory presented and were taken from French standardized naming test D080 (Deloche and Hannequin, 1997). The run consisted of five task conditions of sentence generation performed in the auditory modality (8 stimuli/condition, 40 words in total) and the inter-stimulus intervals (ISI) of 5 s intended to provide enough time to generate sentences. The run also included five control periods (non-generation) to control for auditory activations during which a pseudoword was played eight successive times, with 5 s ISI. The participants were asked not to talk covertly after hearing the pseudoword and just listen to it. The run also included five rest blocks with a fixation cross displayed for 10 s, placed directly after the generation blocks for the hemodynamic response to come down. Participants were told just to fixate the cross. The lineup of conditions was Task (Generation), Rest, and Control. The run took 7.3 min.

#### Recognition of Items Stimuli and Task (RECO)

In the second run, the participants were shown pictures on the screen. They were asked to recognize and respond whether they heard the names of the objects in the images during the GE run. The task had an event-related design, and it included pictures of the words participants heard in the previous task, pictures of the new objects, control images, and rest condition. Participants responded by pressing the buttons on the response box placed in their dominant hand. The stimuli included 40 images of the words participants heard in the GE run (hereafter OLD). For these images, the correct response was pressing the “yes” button. The run also included 40 pictures of the words not heard in the GE run (hereafter NEW). The NEW images also presented the words from the DO80 matched with the words presented in OLD pictures in terms of lexical length and frequency. For the NEW images, the correct answer was pressing the “no” button. The run also contained 40 control images showing the button that needed to be pressed to control motor activations during button pressing. Finally, the run contained 45 null events with a fixation cross. The conditions were presented in a pseudo-randomized order, and the ISI was 2.5 s. Hence all events were displayed for 2.5 s. The duration of this run was 6.8 min. The modality change between the sentence generation and recognition task was employed to impose the access to episodic memory and hence the activation of hippocampal structures.

#### Recall Stimuli and Task (RA)

In the third run, participants again heard the words they heard during the GE run, and they were asked to recall the sentences they previously generated and covertly repeat them. This block-based paradigm contained five tasks (8 stimuli/condition, 40 words in total) and five control (fixation cross displayed for 10 s) conditions. The duration of this task was 4.17 min.

### MR Acquisition

Functional MRI experiments were performed at the IRMaGe MR facility in our clinical facility. MR images were acquired with a whole-body 3T MR Philips imager (Achieva 3.0T TX Philips, Philips Medical Systems, Best, NL) with a 32-channel head coil for all participants. For the functional scans, the manufacturer-provided gradient-echo/T2* weighted EPI method was used. Forty-two adjacent axial slices parallel to the bicommissural plane were acquired in sequential mode (3 mm thickness, TR = 2.5 s, TE = 30 ms, flip angle = 82°, in-plane voxel size = 3 × 3 mm; field of view = 240 × 240 × 126 mm; data matrix = 80 × 80 pixels; reconstruction matrix = 80 × 80 pixels). In addition, for each participant, a T1-weighted high-resolution three-dimensional anatomical volume was acquired by using a 3D T1TFE (field of view = 256 × 256 × 160 mm; resolution: 1 × 1 × 1 mm; acquisition matrix: 256 × 256 pixels; reconstruction matrix: 256 × 256 pixels).

### Data Processing

#### Behavioral Analyses of the RECO Task

Based on the responses during the RECO run, we calculated behavioral performances for the memory recognition task. Statistical analyses were performed using Jamovi statistical software [The jamovi project (2020). jamovi (Version 1.6)^1^ ]. The Mann–Whitney *U* test was used to test the differences in the responses between LTLE patients and HC. To compare an individual patient’s score with the score of the HC group, we used the modified Crawford test (Crawford and Garthwaite, 2002) via Singlim software^2^.

#### Functional MRI Analyses

##### Preprocessing Steps

The preprocessing was performed using SPM12 (Welcome Department of Imaging Neuroscience, London, United Kingdom^3^) running under Matlab R2019b (Mathworks Inc., Natick, MA, United States) using the standard routines. All images were realigned to correct the head motion, time-corrected with the mean image as the reference slice, spatially normalized to MNI (Montreal Neurological Institute) space, and then spatially smoothed with an 8 mm FWHM (Full Width at Half Maximum) Gaussian kernel. The T1-weighted anatomical volume was co-registered to the mean image created by the realignment procedure and was normalized within the MNI (Montreal Neurological Institute) space. The anatomical normalization parameters were subsequently used for the normalization of functional volumes.

##### Functional MRI Statistical Analyses

Since the clinical utility of an fMRI protocol is highly associated with its ability to identify the dominant hemisphere for a specific function and the network of regions engaged in that function (Benjamin et al., 2020), the analyses employed in this study aimed to determine the regions active during interactive language and memory GE2REC tasks. Nevertheless, other analyses could also be applied to the fMRI data obtained with this protocol, such as functional connectivity (Banjac et al., 2021) that can also contribute to the presurgical evaluation of epilepsy patients (Foesleitner et al., 2021).

Sentence generation and Recall runs were analyzed as a block design, while Recognition run was analyzed as an event-based design. Statistical parametric maps were generated from linear contrasts between the HRF parameter estimates for the different experimental conditions. The whole-brain effects of interest were firstly evaluated at an individual level (first-level) to assess: (1) effect of language by comparing sentence generation with the baseline; (2) effects of memory recognition by comparing correctly recognized items with the baseline; and (3) effects of memory recall by comparing sentence repetition with the baseline. Six movement parameters obtained by realignment corrections were included as noise (regressors of non-interest).

For the second-level group analyses, individual contrasts were entered into a one-sample *t*-test, and activations were reported at a *p* < 0.05 significance level with the FWE correction (*T*_GE_ > 6.89 for sentence generation, *T*_RECO_ > 7.03 for recognition, and *T*_*RA*_ > 6.85 for recall task) with a threshold of 5 voxels (*k* > 5) for all effects. Moreover, we also repeated the second-level group analyses at a more permissive threshold (*p* < 0.001 uncorrected) to test if the activation can be identified in regions expected to be engaged in language and memory processing by previous studies and models. An additional reason for threshold lowering is that one of the hub regions of the LMN, the hippocampus, and mesial temporal structures in general, can be affected by geometric distortions and signal loss (Powell and Duncan, 2005; Haag and Bonelli, 2013; Buck and Sidhu, 2020).

To validate the ability of the protocol to activate the expected LMN robustly, maps provided by the GE2REC were compared with the maps obtained via Neurosynth for language and memory^4^ (Yarkoni et al., 2011) in terms of AAL regions coverage (Tzourio-Mazoyer et al., 2002). The procedure is explained in detail in the Supplementary Material.

Differences between LTLE and HC were tested to explore neuroplasticity from a more fundamental perspective. Therefore, the same first-level analyses were first performed for HC. Then the individual contrasts of LTLE and HC were entered into a two-sample *t*-test to perform third-level group analyses. Since there was a significant age difference between LTLE and HC, we added age as a covariate. Considering that the addition of the regressors can decrease statistical power (Lazar, 2008), activations were reported at a lower threshold (*p* < 0.001 uncorrected) *T* > 3.35 for all tasks and a threshold of 5 voxels (*k* > 5).

For the two case studies, first-level analyses were performed before and after surgery. Resection volumes were defined on normalized T1 images using manual contouring in MRIcron^5^. As in the case of second-level group analyses, the activations were reported at a *p* < 0.05 significance level with the FWE correction (*T* > 4.59) and also at a more permissive threshold (*p* < 0.001) for all tasks with a threshold of 5 voxels (*k* > 5). Patients and HC were compared using a two-sample *t*-test.

In order to explore the informativeness of the preoperative LMN map obtained by the GE2REC protocol for predicting neuropsychological outcomes after surgery, we calculated the percentage of preoperative LMN that was resected (You et al., 2019). This was done for patients whose postoperative images we had available. We binarized the activation maps obtained for GE, RECO, and RA tasks at *p* < 0.001, uncorrected, and *k* > 5 thresholds for each patient separately. We then added these three maps to obtain the individual LMN map. These maps were then overlapped with the anatomic resection masks (masks were normalized to MNI space). The sum of voxels activated in the resected area was divided by the total number of voxels in their presurgical LMN maps for each patient.

##### Hemispheric Lateralization and Reorganization

We assessed the lateralization index (LI) of activations using the bootstrap method of the SPM LI toolbox (Wilke and Lidzba, 2007). This method was chosen because it is threshold-independent, robust, and resistant to outliers. We calculated general LIs for frontal and temporal cortices and regions of interest (ROI). The ROI LIs were calculated to evaluate the efficiency of cortical organization and reorganization of TLE patients (see the following subsection). We employed specific ROIs instead of the whole lobe LIs so that the obtained results could be interpreted in terms of specific processes. Although many LMN regions are essential for proper cognitive functioning, we focused on those considered hubs. Specifically, we included inferior frontal orbitalis, triangularis, and opercularis engaged in multiple language processes (e.g., semantic and syntactic) and performing unification and integration (Hagoort, 2016). The middle temporal gyrus was included as a part of the lexico-semantic network (Binder and Desai, 2011; Price, 2012; Middlebrooks et al., 2017; Hertrich et al., 2020), while the inferior parietal cortex was included for its engagement in semantic and control processing (Baldo and Dronkers, 2006; Bzdok et al., 2016; Wen et al., 2018). Finally, the hippocampus was included since it plays a unifying role by binding the features into a coherent representation and supporting flexible cortical retrieval (Reagh and Ranganath, 2018; Cooper and Ritchey, 2019), and was proposed to be the link between language and memory (Tracy and Boswell, 2008; Duff and Brown-Schmidt, 2012). ROIs were anatomically defined using the WFU pickatlas toolbox (Maldjian et al., 2003, 2004) and the AAL atlas (Tzourio-Mazoyer et al., 2002).

According to the more fundamental aim of this paper, the potential reorganization according to status (LTLE or HC) was tested by exploring the differences in LIs between groups using the Man–Whitney *U* test and the effects of task and lobe, using the Friedman test for repeated measurements with Durbin-Conover test for pairwise comparisons. We used the modified Crawford test to compare an individual patient’s LIs with the HC group (Crawford and Garthwaite, 2002). To explore the potential utility of the GE2REC protocol in presurgical evaluation, we explored the hemispheric lateralization in patients with and without postsurgical cognitive decline. We highlight that this data is only illustrative and can serve to formulate hypotheses for future studies since there were not enough participants to perform appropriate statistical analyses.

Since the hippocampal region is of particular interest for patients with LTLE, we specifically calculated the difference between LTLE and HC in the distribution of hippocampal lateralization using the Chi-square test. LIs higher than 0.2 were considered as left-lateralized, and those below, as bilateral to right (Seghier, 2008). We grouped bilateral and right lateralization due to the reduced number of participants, thus having left and non-left lateralizations.

##### The Efficiency and Clinical Correlates of Language-and-Memory Reorganization

To determine the efficiency of potential reorganization, we correlated the LIs-ROIs with either RECO performances or neuropsychological scores. To explore clinical characteristics associated with potential reorganization, we correlated the LIs with the clinical characteristics of TLE patients. The results were FDR corrected for multiple comparisons.

## Results

### Behavioral Results of the RECO Task

Overall, LTLE had lower% of correct responses (%CR) than HC (*U* = 105, *p* = 0.046) and were slower (RTs) than HC (*U* = 39, *p* < 0.001). Details regarding correct responses are presented in Supplementary Table S1. Patients and HC were comparable in terms of gender ratio (χ^2^ = 0.67, *p* = 0.413), but differed with regard to age (*U* = 20, *p* < 0.001). Therefore, we performed a one-way rank analysis of covariance. A non-parametric version was performed since our data did not meet ANCOVA normality assumptions. When age was introduced as a covariate, the LTLE patients did not differ significantly from HC regarding correct responses [*F*(1,35) = 0.01, *p* = 0.921] and reaction time [*F*(1,35) = 3.16, *p* = 0.084].

### Functional MRI

#### Left Temporal Lobe Epilepsy Group Analysis

Figure 1B shows results obtained for the LTLE group. In Supplementary Figure S1, we illustrate the variation of activation across patients, and Supplementary Table S2 provides the mean SD for each AAL region. Hereafter, we present activations for each task resulting from the second-level group analysis in LTLE patients.

***Sentence generation*** activated a wide fronto-temporal network, including bilateral temporal and predominantly left frontal regions (Figure 1B1 and Supplementary Table S3). During this task, left inferior frontal and middle temporal and bilateral superior temporal cortices were activated. Bilateral but predominantly right cerebellar activation mainly of the lobule 6 and Crus 1 was obtained. Left hippocampal and parahippocampal activation was observed at a lower *p*-value (*p* < 0.001).

***Recognition*** recruited a network that included the bilateral fusiform, occipital and inferior temporal, bilateral cingulum, and left superior frontal cortices, as well as left inferior parietal and left hippocampus (Figure 1B2 and Supplementary Table S4). Activation of the right hippocampus and inferior parietal lobule was also detected at a lower *p*-value (*p* < 0.001).

***Recall*** activated a network that included bilateral superior frontal and left inferior frontal cortices, left middle and bilateral superior temporal cortices (Figure 1B3 and Supplementary Table S5). Bilateral occipital and left hippocampal activation was obtained at a lower *p*-value (*p* < 0.001).

#### Correspondence Between Language-and-Memory Network Networks

**FIGURE 2:**
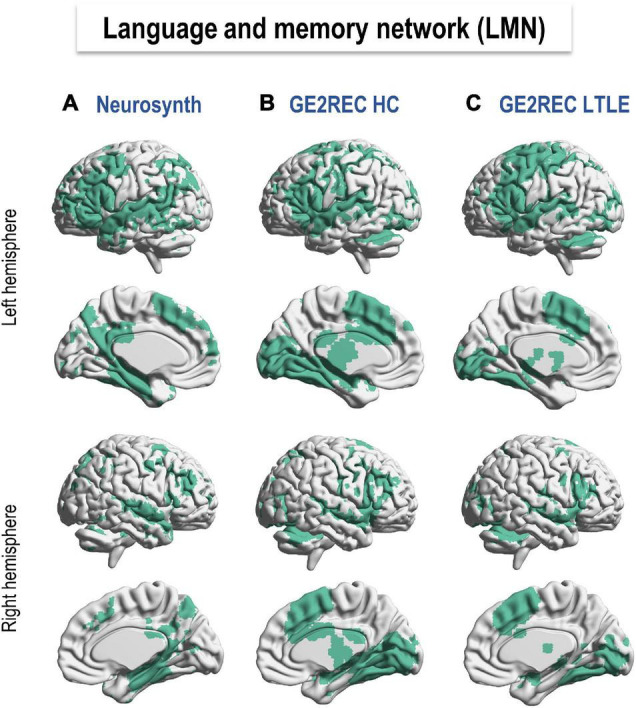
Language and memory networks (LMN) resulting from **(A)** Neurosynth database (http://neurosynth.org; Yarkoni et al., 2011), **(B)** GE2REC in healthy participants, and **(C)** LTLE patients. Specifically, a search for terms *language and memory* in the Neurosynth database yielded 1101 and 2744 studies, respectively. Maps were binarized and added up. GE2REC maps were based on activations provided by the second-level group analyses for HC (*N* = 19) and LTLE (*N* = 18) by all three tasks together. A less permissive threshold (*p* < 0.001 and *k* > 5) was used to binarize GE2REC activation given the limited number of participants compared to the number of meta-analyses and participants in Neurosynth. The LMN correspondence between GE2REC and Neurosynth is reported in Supplementary Table S6 in terms of AAL regions (Tzourio-Mazoyer et al., 2002). LTLE, left temporal lobe epilepsy; HC, healthy controls.

Figure 2 shows the LMN provided by Neurosynth meta-analysis (Figure 2A) and GE2REC protocol in HC and LTLE (Figures 2B,C). Details on activated regions are presented in Supplementary Table S6. This comparison allowed us to claim that the LMN can be robustly activated using the three GE2REC runs. However, some differences with Neurosynth maps were noted in both HC and LTLE. For instance, GE2REC recruited less left prefrontal, left angular and parietal lobule; GE2REC recruited more bilateral supplementary motor area (SMA), insula, occipital cortices, subcortical structures, and cerebellum in both HC and LTLE (see Supplementary Material). Several regions were common only to Neurosynth and GE2REC in HC, such as the superior temporal pole, middle temporal gyrus, and bilateral hippocampi.

#### Language-and-Memory Network Surgical Resection

For patients who underwent surgery and whose postsurgical anatomical images were available (*N* = 7), we calculated the percentage of preoperatively activated LMN resected in the surgery. Patients with greater postoperative cognitive decline (*N* = 4) had on average more than 10% of the activated LMN surgically removed, while those with the reduced decline (*N* = 3) had less than 10% (see Table 1). Nevertheless, at an individual level, we observed that 2/4 of patients with greater cognitive decline had a lower percentage of LMN resected during surgery than those presenting a reduced decline. Still, patients presenting the most important percentage of LMN resection showed a greater cognitive decline after surgery.

#### Hemispheric Lateralization and Group Differences

**FIGURE 3:**
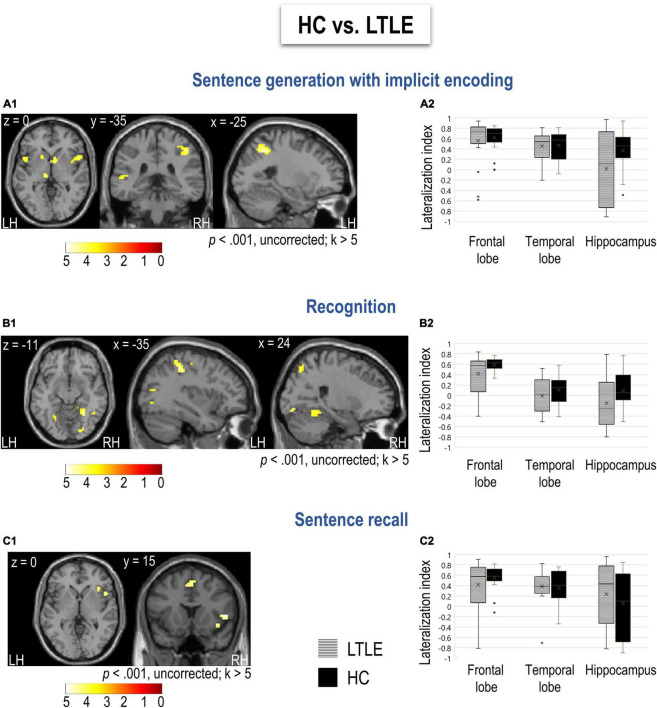
Differences between LTLE and HC for sentence generation, recognition, and recall. **(A1–C1)** Functional maps for HC > LTLE obtained for each task (sentence generation, recognition, and recall, respectively) at a threshold of *p* < 0.001 and *k* > 5. Activations were projected onto 2D axial, coronal, and sagittal slices. The color scale indicates the *T* value. No significant difference was obtained for LTLE compared to HC. **(A2–C2)** Distribution of lateralization indices calculated for frontal and temporal lobes as well as the hippocampus, resulting from each task in the group of LTLE and HC. The mean of each lateralization index distribution is indicated with an x sign and the median with a bar. LH, left hemisphere; RH, right hemisphere; LTLE, left temporal lobe epilepsy patients; HC, healthy controls.

Figures 3A2,B2,C2 and Supplementary Table S7 show lateralization indices for LTLE and HC for the three tasks. Man–Whitney *U* test showed that groups did not differ significantly in terms of tasks, neither for frontal (GE: *U* = 145, *p* = 0.438, RECO: *U* = 138, *p* = 0.323, RA: *U* = 167, *p* = 0.903) nor for temporal lateralization (GE: *U* = 167, *p* = 0.903, RECO: *U* = 135, *p* = 0.274, RA: *U* = 163, *p* = 0.808). Regarding the hippocampal structure, although Man–Whitney *U* test did not show significant differences between two groups (GE: *U* = 124, *p* = 0.158, RECO: *U* = 112, *p* = 0.073, RA: *U* = 139, *p* = 0.331), by categorizing participants based on their lateralization, we observed that compared to HC, LTLE patients show more frequently bilateral to right lateralization of hippocampus during generation task (χ^2^ = 4.68, *p* = 0.031). No such differences were found for the other tasks (RECO: χ^2^ = 3.34, *p* = 0.068; RA: χ^2^ = 0.67, *p* = 0.413).

Friedman test for repeated measurements showed a significant effect of task on the lateralization of temporal lobe (χ^2^ = 12.4, *p* = 0.002), but not frontal (χ^2^ = 1.44, *p* = 0.486) in LTLE. Specifically, during recognition task temporal lobe was less left lateralized than it was during sentence generation (*D* = 4.01, *p* < 0.001) and recall task (*D* = 3.2, *p* = 0.003). This was comparable to HC that also showed the effect of task in lateralization the temporal (χ^2^ = 10.8, *p* = 0.004) but not frontal lobe (χ^2^ = 4.11, *p* = 0.128). In HC, similarly to LTLE, temporal lobe was less left lateralized than during generation (*D* = 3.74, *p* < 0.001) and recall task (*D* = 2.43, *p* = 0.020). In general, in LTLE temporal lobe was less left lateralized than frontal lobe for recognition task (χ^2^ = 5.56, *p* = 0.018), but not for sentence generation (χ^2^ = 2, *p* = 0.157) and recall task (χ^2^ = 0.889, *p* = 0.346). However, in HC temporal lobe was less lateralized to left than frontal lobe during all the tasks (GE: χ^2^ = 4.26, *p* = *0.039*, RECO: χ^2^ = 8, *p* = 0.005, RA: χ^2^ = 6.37, *p* = 0.012).

Table 3 presents hemispheric lateralization of the frontal and temporal lobes and the hippocampus, cumulatively for all three tasks together for patients with greater and reduced postsurgical cognitive decline. Although only descriptive, patients with greater cognitive decline showed more frequently left-lateralized frontal lobe activations. There was an absence of right-lateralized frontal activation in this group, which was identified in the group with reduced cognitive decline. Both groups of patients showed similar lateralization of the temporal lobe. However, data indicate that patients with the greater cognitive decline most frequently showed left-lateralized hippocampus activation, while those with reduced decline frequently showed right lateralization of this structure.

**TABLE 3:** Descriptive statistics of patients with greater cognitive decline after surgery (GD, *N* = 4) and those with the reduced decline (RD, *N* = 3).

	Lateralization								
	Frontal			Temporal			Hippocampus		
Group	L	B	R	L	B	R	L	B	R
GD (*N* = 4)	2.33 (0.82)	0.67 (0.82)	0 (0)	2 (0.89)	0.5 (0.55)	0.5 (0.55)	1.8 (0.98)	0.5 (0.84)	0.67 (0.82)
RD (*N* = 3)	1.75 (0.96)	0.25 (0.5)	1 (1.15)	2 (0.82)	0.5 (0.58)	0.5 (0.58)	0.75 (0.96)	0.5 (1)	1.75 (0.96)

*Lateralization refers to the count of left (L), bilateral (B), and right (R) hemispheric dominance across three GE2REC tasks.*

#### The Efficiency of Language-and-Memory Reorganization

We explored the cognitive efficiency of functional organization and reorganization by correlating lateralization of selected language and memory ROIs with behavioral and cognitive scores. The following results survived corrections for multiple comparisons. In LTLE, AMI scores were negatively correlated with LI of the inferior parietal region during sentence generation (*r*_s_ = –0.7, *p* = 0.003, *p*_adj_ = 0.018), indicating that higher AMI scores were associated with greater right lateralization of this region. Semantic fluency scores were positively correlated with lateralization of inferior frontal pars orbitalis (*r*_s_ = 0.65, *p* = 0.005, *p*_adj_ = 0.03) for sentence generation, as an increase in semantic fluency scores was associated with greater left lateralization of this ROI. There were no significant correlations between clinical variables and LIs after correction for multiple comparisons.

### Left Temporal Lobe Epilepsy vs. Healthy Controls Differences

Since LTLE and HC were comparable in terms of gender but not age, we controlled for the effect of age in all analyses. First, there were no regions more activated in LTLE than HC. Therefore, results hereafter show regions significantly more activated in HC than in LTLE. Specifically, sentence generation revealed more activation of bilateral inferior frontal opercular, parietal, and left superior temporal cortices in HC (Figure 3A1 and Supplementary Table S8). Recognition activated more the bilateral superior parietal, occipital, fusiform, and lingual gyri in HC (Figure 3B1 and Supplementary Table S9). Finally, we obtained more activation of the right inferior pars opercularis and insula during the recall in HC (Figure 3C1 and Supplementary Table S10).

### Case Studies

P1 and P2 were comparable regarding the lateralization of the epileptogenic network. They both showed hippocampal atrophy, same handedness, and education level and were both males. Moreover, they were both seizure-free after surgery (Engel class Ia; Engel et al., 1993; Engel, 2014; Zentner, 2020). However, they were different in terms of age of seizures onset, epilepsy duration, seizure frequency, and the number of antiepileptic drugs, as well as postsurgical cognitive outcome (see Table 2).

#### Patient 1

##### Neuropsychological Scores and Behavioral Performance

**FIGURE 4:**
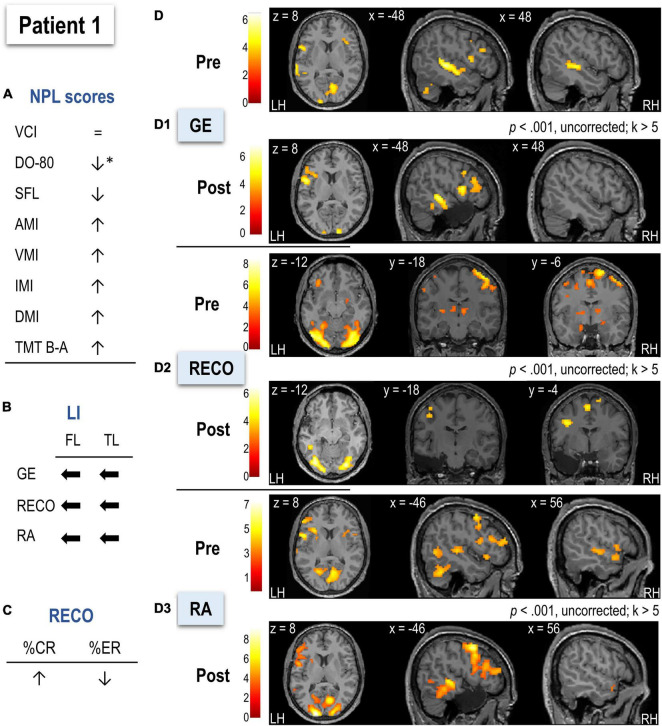
Illustration of the neuropsychological and language scores **(A)**, LIs **(B)**, behavioral performance for recognition (RECO) **(C)**, and functional MRI activation **(D)** in Patient 1 (P1) before and after surgery. Arrows in **(A–C)** indicate the direction of change (increase, decrease, shift to left or right) after surgery. Arrows in **(A)** marked with * indicate a significant score decrease (using a 90% confidence interval). **(D1–D3)** Functional maps for each task (sentence generation, recognition, and recall, respectively) at a threshold of *p* < 0.001, uncorrected, and *k* > 5. Activations were projected onto the normalized anatomical image of P1 before and after surgery. The color scale indicates the T value. NPL, neuropsychological and language assessment; LI, lateralization index; GE, sentence generation with implicit encoding; RECO, recognition of items; RA, recall; LH, left hemisphere; RH, right hemisphere.

Neuropsychological and language evaluation of P1 before surgery (Figure 4A and Table 2) showed no cognitive deficit. Postoperatively, anterograde memory indices slightly increased, and naming performance significantly decreased (using a 90% confidence interval) but remained within norms. Descriptive statistics showed that P1’s RECO performance (based on %CR and %ER) slightly increased after surgery (Figure 4C and Table 2).

##### Functional MRI

The postoperative LMN was more activated in the left frontal and temporal lobes than preoperative (see Figures 4B,D and Table 2).

###### Sentence Generation

In pre-surgery, the highest activation was noticed for left middle and right superior temporal, left cuneus, bilateral lingual gyri, and occipital cortices (Figure 4D1 and Supplementary Table S11). Compared to HC (Supplementary Table S12), P1 showed more significant activation of the right cingulate, supramarginal gyrus, left hippocampus, and inferior temporal gyrus. After surgery (Supplementary Table S13), P1 activated a predominant left-lateralized network encompassing mainly the SMA, superior and middle temporal cortices, and inferior frontal operculum. Activations of left superior and middle temporal cortices were stronger in P1 than in HC (Supplementary Table S14). The comparison of P1 to HC in terms of LIs did not reveal significant differences.

###### Recognition

Before surgery, P1 activated bilaterally left frontal and temporal lobes (Figure 4D2 and Supplementary Table S15). The highest activations were observed in inferior regions such as occipital, fusiform, and lingual cortices, SMA, precentral, and left parietal inferior. Activation of bilateral but predominantly to the right hippocampi was also observed. Compared to HC, P1 relied more on right hemisphere regions such as SMA, precentral, temporal pole, postcentral, and bilateral hippocampi (Supplementary Table S16). In terms of lateralization, P1 was more bilateral for frontal regions than HC (*t* = –5.07, *p* < 0.001). After surgery, the recognition network was mostly left-lateralized and included mainly occipital and parietal superior cortices and bilateral fusiform gyrus (Supplementary Table S17). Postsurgical lateralization of frontal and temporal regions was not significantly different from HC (Table 2).

###### Recall

Before surgery, P1 showed bilateral activation but predominantly to the left, including left frontal inferior operculum, posterior middle and inferior temporal cortices, left SMA and supramarginal gyrus, and the right superior temporal pole. P1 activated the right angular gyrus more than HC (Figure 4D3 and Supplementary Table S19). The hippocampal activation was not observed at the applied threshold, but LIs calculated across thresholds suggested a right predominance of this region (Table 2). After surgery, P1 showed a predominantly left-lateralized network with the highest activation for middle and superior occipital, middle, and inferior frontal as well as middle and inferior temporal cortices (Supplementary Table S21). Compared to HC, P1 also relied more on a left temporo-occipital network (Supplementary Table S22). There was no significant difference from HC regarding LIs before or after surgery (Table 2).

After surgery, 3.98% of the LMN P1 activated in preoperative assessment was removed.

#### Patient 2

##### Neuropsychological Scores

**FIGURE 5:**
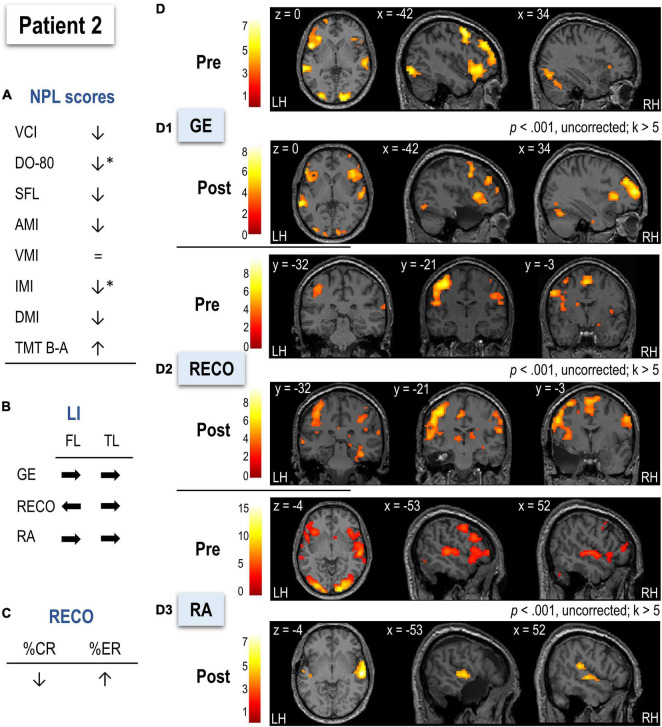
Illustration of the neuropsychological and language scores **(A)**, LIs **(B)**, behavioral performance for recognition (RECO) **(C)**, and functional MRI activation **(D)** in Patient 2 (P2) before and after surgery. Arrows in **(A–C)** indicate the direction of change (increase, decrease, shift to left or right) after surgery. Arrows in **(A)** marked with * indicate a significant score decrease (using a 90% confidence interval). **(D1–D3)** Functional maps for each task (sentence generation, recognition, and recall, respectively) at a threshold of *p* < 0.001, uncorrected, and *k* > 5. Activations were projected onto the normalized anatomical image of P2 before and after surgery. The color scale indicates the *T* value. NPL, neuropsychological and language assessment; LI, lateralization index; GE, sentence generation with implicit encoding; RECO, recognition of items; RA, recall; LH, left hemisphere; RH, right hemisphere.

Neuropsychological and language evaluation of P2 before surgery (Figure 5A and Table 2) showed pathological scores for mental flexibility. Postoperatively, immediate memory and naming scores decreased (using a 90% confidence interval), and the latter showed pathological value, while auditive and delayed memory only slightly decreased. Descriptive statistics showed that P2’s RECO performance (based on %CR and %ER) decreased after surgery (Figure 5C and Table 2).

##### Functional MRI

The postoperative LMN showed greater implication of the right frontal and temporal hemispheres than the preoperative (see Figures 5B,D and Table 2).

###### Sentence Generation

In pre-surgery, P2 mainly activated left superior, middle, and inferior frontal cortices, left superior temporal pole, and bilateral temporal superior cortices together with bilateral temporo-occipital cortices (Figure 5D1 and Supplementary Table S23). Compared to HC (Supplementary Table S24), P2 relied more on left occipital cortices, left insula, and bilateral middle frontal and superior parietal cortices. The hippocampal activation was not observed at the applied threshold, but LIs calculated across thresholds suggested left predominance (Table 2). After surgery, P2 activated a network composed of bilateral superior and inferior frontal regions with slight left predominance, as well as bilateral superior temporal and occipital cortices (Supplementary Table S25). There were no significant differences between P2 and HC regarding LIs before or after surgery (Table 2).

###### Recognition

Before surgery, P2 showed activation mainly in bilateral frontal and occipital cortices, bilateral superior parietal regions, and right anterior hippocampus (Figure 5D2 and Supplementary Table S27). The bilateral predominantly left posterior and inferior temporal activation was also observed. In terms of lateralization, P2 was more bilateral for frontal regions than HC (*t* = –3.82, *p* < 0.001; Table 2). After surgery, a larger recognition network was composed of predominantly left prefrontal, superior, and middle frontal cortices, bilateral occipital regions, and predominantly right inferior and posterior superior temporal cortices with the posterior right hippocampus (Supplementary Table S29). Frontal and temporal postsurgical lateralization was not significantly different from HC (Table 2).

###### Recall

Before surgery, P2 activated a predominantly left-lateralized network, including frontal superior and inferior cortices, occipital regions, and superior temporal and parietal cortices (Figure 5D3 and Supplementary Table S31). P2 activated more right parietal superior and bilateral occipito-temporal regions than HC (Supplementary Table S32). The hippocampal activation was not observed at the applied threshold, but LIs calculated across thresholds suggested left predominance (Table 2). There was no significant difference from HC in terms of LIs before surgery (Table 2). After surgery, P2 activated a network mainly composed of bilateral but predominantly right-lateralized temporal cortices (S33). Compared to HC in terms of LIs, P2’s frontal and temporal regions were significantly reorganized to the right hemisphere (frontal: *t* = –3.21, *p* < 0.05; temporal: *t* = –2.32, *p* < 0.05; Table 2).

After surgery, 5.25% of the LMN P2 activated in preoperative assessment was removed.

## Discussion

In this study, we applied the GE2REC protocol previously validated in healthy participants (see Banjac et al., 2020, 2021) to map LMN underlying the language and declarative memory interaction in a group of LTLE patients, candidates for surgery. Functional interactivity of the protocol is provided since each task demands both functions (see Banjac et al., 2021), and the three runs are interrelated. Specifically, during GE, participants perform word recognition, lexico-semantic search, and sentence production, as well as implicit encoding and contextual binding, particularly related to episodic memory (Yonelinas et al., 2019). RECO is based on object naming and memory recognition. Finally, RA engages word recognition, lexico-semantic search that triggers episodic retrieval and concept access, sentence recall, and sentence production (Banjac et al., 2021). In terms of memory, this protocol assesses declarative memory since GE2REC task performance engages both episodic and semantic memory, accessing different memory processes throughout the tasks (encoding – retrieval – recall; Burianova et al., 2010; Palacio and Cardenas, 2019; Banjac et al., 2021). In this paper, the generic term *memory* will be used to indicate different types and several memory processes.

The interaction between language and memory is essential for everyday functioning, and that calls for their joint investigation instead of trying to untangle them. This interaction is particularly important in LTLE patients in whom these functions are often imperiled, and their (usually reorganized) representations are intermeshed (Tracy and Boswell, 2008). This study had two objectives. The first one was to explore neuroplasticity of the language and memory network, in line with which we explored the reorganization of LMN in LTLE patients by comparing them with HC. The second aim of this study was to explore, at a preliminary level, the potential clinical benefits of this protocol in patients who undergo surgery. Moreover, to show the robustness of LMN mapping at an individual level, we presented two case report studies in more detail.

According to previous studies, models, and meta-analyses (Vigneau et al., 2006; Spaniol et al., 2009; Price, 2012; Benjamin et al., 2017; Labache et al., 2019; Roger et al., 2020a), the theoretical LMN network would engage an extensive bilateral but predominantly left-lateralized fronto-temporo-parietal network. It would include inferior frontal regions for lexico-semantic search and lexical production, as well as bilateral mesial and lateral (middle and inferior) temporal and parietal cortices, required for language, semantic and episodic memory processes. As illustrated in Figure 2, the GE2REC LMN corresponds to the LMN that emerges from the meta-analysis, although some areas, such as prefrontal and parietal cortices, are less recruited by our protocol. The lack of parietal activation on a group level may be due to the fact that this protocol does not accentuate phonological processing (Cousin et al., 2007; Trébuchon et al., 2013). However, case studies showed the individual level activation of parietal regions (inferior, superior, supramarginal, and angular gyri). This suggests that patients may use different strategies for performing the tasks. Therefore, the advantage of the GE2REC protocol is also its ability to map networks according to strategies employed by TLE patients. Another important benefit of using GE2REC in terms of the activated network is that it succeeds in recruiting some particularly significant regions for language and memory, such as the putamen (Viñas-Guasch and Wu, 2017), the thalamus (Llano, 2016), and the cerebellum (Keren-Happuch et al., 2014; Lævenbruck et al., 2018; Gatti et al., 2021), often “neglected” from the most important neurocognitive models of language and memory. Several temporal regions observed in HC were less recruited by LTLE, probably due to their pathology, especially the left temporal and hippocampi (Thom and Bertram, 2012; Barr, 2015).

In line with our first aim – to understand how the integrative LMN is reorganized in LTLE, we will discuss differences between LTLE and HC based on all three GE2REC tasks and at a global instead of regional level within a meta-networking framework, as proposed by Herbet and Duffau (2020). LMN can indeed be considered as a meta-network as natural communication cannot operate based on one system without the additional supporting systems (Hertrich et al., 2020). Our LTLE patients showed widespread reorganization in the LMN, mainly manifested as a reduced activity of the regions having an integrative role or engaged in cognitive control (Binder and Desai, 2011; Ranganath and Ritchey, 2012; Burianová et al., 2017; Forseth et al., 2018). Specifically, within the semantic network engaged by all three tasks, LTLE patients showed less activation of “convergence” regions (such as inferior parietal and fusiform gyri) and the regions engaged in the control of goal-directed action and information selection (such as dorsomedial and inferior prefrontal cortices) (Binder and Desai, 2011; Forseth et al., 2018). Regarding syntactic processing, LTLE showed weaker activation of the inferior frontal gyrus (IFG), a region that integrates dorsal and ventral streams under the cognitive prefrontal control (Weiller et al., 2016). Within memory networks, LTLE patients showed reduced activity of regions belonging to the posterior medial system (Ranganath and Ritchey, 2012; Palacio and Cardenas, 2019) that serve as an interface between the semantic and episodic system and as an integrator between modalities and subsystems, such as precuneus (Binder and Desai, 2011), angular gyrus (Ranganath and Ritchey, 2012; Seghier, 2013; Humphreys et al., 2021) and thalamus (Wolff and Vann, 2019). The interdependence and overlap of semantic and episodic memory systems have been shown, and cognitive control is one of the underlying processes shared across these systems (Burianova et al., 2010; Vatansever et al., 2021). LTLE indeed activated less the regions of ventral attention or salience network (SAL) and dorsal attention network (DAN). Within the SAL, engaged in coordination of attentional resources, cognitive control, and recruitment of resources to provide responses (Burianová et al., 2017; Hertrich et al., 2020), LTLE showed decreased activation of the insula, anterior cingulate, and SMA. Concerning the DAN, involved in goal-directed and top–down attention (Vossel et al., 2014; Dixon et al., 2017), LTLE showed decreased activation of superior and inferior parietal, pre- and postcentral cortices. The disfunction of these networks, already identified in TLE (Zhang et al., 2009; Burianová et al., 2017), could result from long-term seizure propagation (Burianová et al., 2017). These effects might manifest as poorer coordination of attention and reduced allocation of attention to language and memory processes, leading to weaker activation of regions performing integration within semantic, syntactic, and memory subsystems and between them. Therefore, language and memory deficits observed in TLE (Zhao et al., 2014; Jaimes-Bautista et al., 2015; Tramoni-Negre et al., 2017; Dutta et al., 2018) could be explained by the weaker cross-network interactions and dynamics due to poorer involvement of regions that act as an interface between multiple functional systems (Herbet and Duffau, 2020). The reduced activation of these interface regions might be explained by the weaker activation of attention-control networks in TLE patients.

Although inter-hemispheric network reorganization is a common finding in LTLE (Goldmann and Golby, 2005; Powell et al., 2007; Cousin et al., 2008; Hamberger and Cole, 2011; Bonelli et al., 2012; Sidhu et al., 2013; Baciu and Perrone-Bertolotti, 2015; Torlay et al., 2017; Foesleitner et al., 2021), our patients did not show an evident inter-hemispheric reorganization as revealed by group-level analyses. This result can be explained by the fact that our patients had late age of seizures onset (ASO, see Table 1) generally associated with intra-hemispheric reorganization, compared to patients with early ASO who more frequently show inter-hemispheric reorganization (Baciu and Perrone-Bertolotti, 2015). Additionally, patients were right-handed, less likely to show atypical lateralization (Mazoyer et al., 2014). However, the comparison of LIs calculated at a regional level showed that the lateralization of frontal and hippocampal regions was more variable in LTLE than in HC (Supplementary Table S7). This suggests that GE2REC manages to yield various types of LTLE-related reorganization as suggested by Berl et al. (2014), which, however, could not stand up as a unique pattern at a group level. Additionally, regional level analyses revealed that most patients did not show left hippocampal activation during encoding, suggesting reorganization at this level as reported by previous studies (Sidhu et al., 2013; Dupont, 2015). We also note that regional lateralization tends to change across tasks in both HC and LTLE. This is in line with previous findings suggesting that hemispheric lateralization for language is not a rigid and a unitary construct (Bradshaw et al., 2017, 2019) but varies according to regions and specific processes. Similarly, it was shown that different memory processes and types of stimuli could result in different memory lateralization (Golby et al., 2002; Milian et al., 2015; Andreau and Torres Batán, 2019; Palacio and Cardenas, 2019). One of the advantages of GE2REC is that it includes both verbal and visual material, different language, and memory processes, allowing for comprehensive preoperative screening of regional lateralization.

Regarding cognitive efficiency in LTLE patients, we found that better semantic fluency performance (Godefroy and GREFEX, 2008) was associated with greater left-lateralization of IFG (orbitalis), one of the LMN integrative hubs (Weiller et al., 2016; Banjac et al., 2021). Verbal fluency scores were indeed found to correlate with left IFG activation in LTLE patients suggesting its involvement in the functional integrity of language network in these patients (Bonelli et al., 2011). On the other hand, better memory performance (IMA, Wechsler, 2009) was associated with increased right-lateralization of the inferior parietal lobule. This region is a part of DAN and FPN control networks engaged in the attention and coordination of interaction between networks (Yeo et al., 2011; Vossel et al., 2014; Dixon et al., 2017). Indeed, attention difficulties can influence the auditive memory index (Holdnack and Drozdick, 2010). While the dorsal parts of attention networks usually show symmetrical engagement (Bartolomeo and Seidel Malkinson, 2019), the right lateralization of these regions was more beneficial for our patients. This could be interpreted as a compensatory mechanism of using additional executive resources from the right hemisphere, as observed in older adults (Gertel et al., 2020; Baciu et al., 2021). Taken together, our findings for cognitive efficiency suggest that LTLE have better preoperative cognitive performance if the LMN is relying more on the left hemisphere for integration processes and on the right hemisphere capacities for cognitive control.

Hence, regarding the first aim of this study, group results showed that the LMN of LTLE patients is similar to that found in HC. LTLE patients did not show dramatic inter or intra-hemispheric LMN reorganization, but rather a mix of the two primarily manifested as reduced activation of regions within the control networks and integrative LMN regions. These results suggest the importance of integration and coordination within multiple functional systems, such as the LMN.

Our second aim was to assess preliminary GE2REC potential clinical informativeness for patients who undergo surgery and its robustness at an individual level. We addressed this aim in two ways, first exploring the descriptive results for the LTLE patients that underwent surgery and second by detailed analysis of GE2REC results for P1 and P2.

As previously mentioned, the number of patients who underwent surgery limited the statistical analyses. Nevertheless, in line with the study’s second aim, we presented the descriptive statistics of the patients whose postoperative data were available. LTLE patients from our sample who had greater postsurgical cognitive decline (i.e., significant worsening of scores from two or more neuropsychological tests) were generally older, had later onset of seizures and shorter epilepsy, and took more antiepileptic medication. These clinical characteristics have indeed been related to poorer postsurgical cognitive outcomes (Dulay and Busch, 2012; Dupont, 2015). Our GE2REC group-level descriptive results suggest that these patients have a greater percentage of their preoperative LMN resected during surgery than patients with more reduced cognitive decline. However, these differences appeared mainly because 2/4 of patients with a greater cognitive decline also had a greater percentage of LMN resection. Also, 2/4 patients with greater cognitive decline had a percentage of LMN resection similar to those presenting reduced cognitive decline. Overall, although we provide some paths of data interpretation regarding the resection guidance in TLE patients, these results should be considered at this moment as only preliminary. The cohort is being improved, and data is being diversified and enriched as the final objective is to provide robust, simple, and specific biomarkers to be used by the clinicians, similar to what You et al. (2019) reported. They indicated that the resection of the activated temporal region would predict the postoperative decline for naming. Another critical inquiry in presurgical evaluation is the activation patterns that would suggest a postoperative cognitive decline. Available data (Table 3) implies that patients with exclusively left-lateralized frontal activation and more frequent left-lateralized hippocampal activation could have a higher risk of postoperative cognitive deterioration. We highlight once again that these are merely descriptive data and should be tested further before any firm conclusions, although they correspond well with the previous studies (Richardson, 2004; Bonelli et al., 2010, 2013; Dupont, 2015). Since the level of interest in clinical practice is a patient rather than a group, we will discuss the results of the two case studies in more detail.

Both P1 and P2 had surgery in the dominant hemisphere and were cognitively unimpaired preoperatively for language and memory (Table 2). Hence, according to previous studies, they were at greater risk of postoperative cognitive decline (Strandberg et al., 2017; Busch et al., 2018). Although the surgery was successful for both patients as they became seizure-free, they “paid a different cognitive price” (Baxendale and Thompson, 2018) as P2 showed a greater cognitive decline. Indeed, P1 showed a semantic decline as indicated by the decrease of naming performance even if it remains within norms and a slight decrease of semantic fluency score. On the other hand, surgery in P2 compromised language and declarative memory functioning (semantic and episodic), as reflected by the significant decrease of naming performance with a pathological score, the significant decline of immediate memory score, and the slight decrease of delayed and auditive memory indices. The worse negative cognitive outcome of P2 after surgery can be explained by the late ASO, in agreement with previous findings reporting that seizures occurring later in life are more detrimental for memory and for naming after temporal surgery (Dulay and Busch, 2012; Dupont, 2015). Nevertheless, the early ASO only in P1 cannot account for the decline in naming (even if values are within norms) after surgery. A possible explanation lies in different activation patterns suggesting different strategies employed by these two patients during GE2REC, as suggested by our fMRI results.

Regarding lexico-semantic processing, P1 and P2 showed strong left hemispheric lateralization during the sentence generation and recall tasks requiring significant language-related resources, as determined by the protocol design. Given the planned left-hemispheric surgery with resection of the anterior temporal lobe, a high risk of language impairment in post-surgery was expected (Hermann et al., 1999) and, unfortunately, indeed observed, postoperatively. However, the naming decline was greater in P1 than in P2, which could be explained by resectioning the patients’ LMNs identified preoperatively using GE2REC. Although the resected areas of P1’s and P2’s preoperative LMN included superior temporal region and temporal pole, in P1, it also included mesial temporal regions. Left mesial regions were engaged in sentence generation and recognition preoperatively in P1 (Supplementary Tables S11, S15) but were removed in surgery. These mesial activations of P1 were even greater than healthy controls (Supplementary Tables S12, S16). This mesial temporal implication aligns with previous studies showing the mesial temporal contribution to word retrieval during picture naming and word fluency tasks (Bonelli et al., 2011; Hamamé et al., 2014). On the other hand, P2 showed greater right-hemispheric involvement after surgery, as revealed by sentence generation and recall tasks, in line with previous findings on reorganization patterns (Bonelli et al., 2012; Foesleitner et al., 2021). In the P2 case, the reorganization was not cognitively efficient, possibly related to weaker coupling between left and right frontal regions (Bonelli et al., 2012; Foesleitner et al., 2021) probably induced by late ASO. Indeed, seizures occurring late in life do not provide sufficient time for an efficient reorganization. Consequently, the right-hemisphere activation may still be an “unbeaten path” and cognitively inefficient (Dulay and Busch, 2012; Dupont, 2015).

Focusing now on specific episodic memory processes assessed by the GE2REC protocol, we first noted no memory impairment in P1 according to scores. On the contrary, P2 showed a significant decrease in immediate memory index, indicating encoding difficulties (Drozdick et al., 2018). In addition, the slight decrease in auditory and delayed memory indices also suggested retrieval dysfunction, possibly in close relation with the encoding decline. Moreover, RECO performance in P2 decreased, while in P1, the performance was enhanced. To understand these differences between P1 and P2, we discuss the lateralization of memory structures as a predictor of the postsurgical memory outcome (Richardson, 2004; Milian et al., 2015). Based on the functional adequacy model (Richardson, 2004; Bonelli et al., 2010, 2013; Dupont, 2015), we expect that patients who show the greater function of the pathological hippocampus across different memory processes face a higher risk of postoperative memory decay. Due to GE2REC advantage of recruiting several memory processes, we observe that P1 showed left lateralization for encoding and consistent right lateralization for retrieval. In parallel, P2 showed left lateralization for encoding and mixed left and right lateralization for retrieval, suggesting that resection surgery of functional hippocampus, although pathological, may increase the risk of memory decline after surgery (Bonelli et al., 2010). Even though P2 showed postoperatively an activation of the right hippocampus during recognition, this single activation is not cognitively efficient, judging by the reduction of correct responses and neuropsychological memory scores after surgery.

Based on the presented findings, we believe that the fMRI GE2REC protocol has several advantages for the preoperative assessment of TLE patients who are candidates to surgery: (1) it robustly assesses the expected LMN in LTLE patients at a group level and provides individual-level information regarding functional localization of language and declarative memory processes; therefore, it can reveal strategies and multiple reorganization patterns corresponding to different processes explored by the protocol; (2) it recruits subcortical and cerebellar regions within the LMN, regions often “neglected” from the classical models, despite their crucial role in language and declarative memory function and reorganization (Llano, 2016; Viñas-Guasch and Wu, 2017; Lævenbruck et al., 2018; Gatti et al., 2021); (3) it assesses in an intermeshed fashion, several language (mainly lexico-semantic and syntactic in comprehension and production) and memory (encoding, recognition and recall) processes, as well as the two types (semantic, episodic) of long-term memory, based on both visual and auditory modalities; notably, the protocol design makes the interconnection between these processes both *horizontally* (within-run) and *vertically* (between-runs); (4) it provides significant information on the lateralization patterns; (5) it activates variably the medial temporal structures including the hippocampus, according to tested processes by each task, and even if the activation of medial temporal structures is weaker than of other regions; this is an important aspect to be underlined, given the difficulties to design an fMRI protocol that activates these structures (Powell and Duncan, 2005; Haag and Bonelli, 2013; Buck et al., 2020); (6) it provides the possibility to assess the cognitive efficiency of activated patterns, by correlating cognitive scores with cerebral fMRI activity and derived lateralization indices.

Independently of its application in TLE patients, in comparison to other standard tasks (such as list reading, for example), GE2REC also has an *ecological* dimension by including a more natural, unintentional, or *everyday life*-like sequence of situations, particularly important for memory functioning (Helmstaedter et al., 1998; Strandberg et al., 2017). Undeniably, this protocol is not as *ecological* as some previously proposed (Bohil et al., 2011; Neri et al., 2021) due to limitations imposed by the clinical setting, hospital equipment, and patient abilities.

If GE2REC protocol has advantages, it also has some general limitations: (1) only RECO behavioral performance can be evaluated, while the overt speech required for GE and RA performances could not be used in the magnet because of the movement artifacts, particularly significant in patients; (2) the duration of tasks could have been longer in order to improve the signal-to-noise ratio mainly for the medial temporal regions; however, a longer protocol was difficult to implement given that the presurgical assessment already includes numerous examinations (MRI, MRI-DTI, PET, neuropsychological assessment) and the total exam duration would be too long for patients; nevertheless, to palliate this limitation, GE2REC recruits several memory types and processes, which increases the likelihood of medial temporal and hippocampal activation; (3) as an important notice, performing this protocol requires a certain level of memory conservation, so it cannot be used in TLE patients with severe memory impairment; (4) although G E2REC allows the integrative assessment of language and memory processes, to evaluate the network properties of LMN, a resting-state fMRI run should be added to this task-based protocol, and functional connectivity analyses should be performed on both types of data (Banjac et al., 2021; Foesleitner et al., 2021); (5) the benefit of GE2REC for the clinical practice in resection planning should be more robustly documented and validated in a larger patient cohort, in association with functional and structural connectivity data.

## Conclusion

We assessed the potential of the fMRI GE2REC protocol to map the interactive LMN in LTLE patients. We presented results suggesting the robustness of this protocol at a group and individual level. As with all fMRI protocols, GE2REC has advantages and several limitations. Therefore, a compromise should be found between advantages and disadvantages, as often required in clinical practice.

## Data Availability Statement

The raw data supporting the conclusions of this article will be made available by the authors, without undue reservation.

## Ethics Statement

The studies involving human participants were reviewed and approved by the Ethics committee for the protection of persons (CPP 09-CHUG-14; MS-14-102). The patients/participants provided their written informed consent to participate in this study. Written informed consent was obtained from the individual(s) for the publication of any potentially identifiable images or data included in this manuscript.

## Author Contributions

SB, ER, EC, and MB: conception and study design. ER, EC, LM, CM, AK, and PK: data acquisition. SB, ER, and EC: statistical analysis. SB, ER, and MB: interpretation of results. SB, ER, EC, CM, AK, LM, PK, and MB: drafting the manuscript work or revising it critically for important intellectual content. All authors: approval of the final version to be published and agreement to be accountable for the integrity and accuracy of all aspects of the work.

## Conflict of Interest

The authors declare that the research was conducted in the absence of any commercial or financial relationships that could be construed as a potential conflict of interest.

## Publisher’s Note

All claims expressed in this article are solely those of the authors and do not necessarily represent those of their affiliated organizations, or those of the publisher, the editors and the reviewers. Any product that may be evaluated in this article, or claim that may be made by its manufacturer, is not guaranteed or endorsed by the publisher.

## Footnotes

1
https://www.jamovi.org

2
https://bit.ly/3e3VI7u

3
http://www.fil.ion.ucl.ac.uk/spm/

4
http://neurosynth.org

5
https://www.nitrc.org/projects/mricron
