# Supplementary material for: Mapping of Language-and-Memory Networks in Patients With Temporal Lobe Epilepsy by Using the GE2REC Protocol
Source: Front Hum Neurosci. 2022 Jan 6;15:752138. doi: 10.3389/fnhum.2021.752138 (PMC8772037; doi:10.3389/fnhum.2021.752138)
Supplement: Supplementary file 1 [file Data_Sheet_1.pdf]

Table S1. Behavioural performance and differences between LTLE and HC during recognition task

		Old items				New items				All items			
		**%CR**	**%ER**	**%NR**	**RT**	**%CR**	**%ER**	**%NR**	**RT**	**%CR**	**%ER**	**%NR**	**RT**
**Mean**	LTLE	72.4	21	6.67	1.08	75.6	13.8	10.7	1.18	74	17.4	8.68	1.13
	HC	71.2	24.9	3.82	0.97	87.5	4.61	8.03	1.12	79.3	14.7	5.91	1.05
**Median**	LTLE	73.8	20	6.25	1.08	81.3	7.5	7.5	1.2	75	16.3	8.13	1.12
	HC	72.5	25	5	0.96	87.5	2.5	7.5	1.13	81.3	13.8	6.25	1.05
**SD**	LTLE	8.81	8.45	4.93	0.1	17.4	14.5	10.8	0.11	8.24	7.31	6.87	0.08
	HC	9.55	9.98	2.41	0.08	7.36	5.42	6.1	0.07	6.54	5.83	3.72	0.05
**Difference**	U	152	126	110.5	74.5	96.5	88	158.5	85.5	105	118	134.5	39
	p	0.572	0.173	0.062	0.003	0.024	0.011	0.713	0.01	0.046	0.112	0.272	< 0.001

Abbreviations: %CR – percentage of correct responses; %ER – percentage of incorrect responses; %NR – Percentage of items without response; RT – reaction time in seconds; LTLE – left temporal lobe epilepsy; HC – healthy controls; The difference in performance between HC and LTLE patients is represented by values of Mann-Whitney *U* tests with the corresponding *p* value.

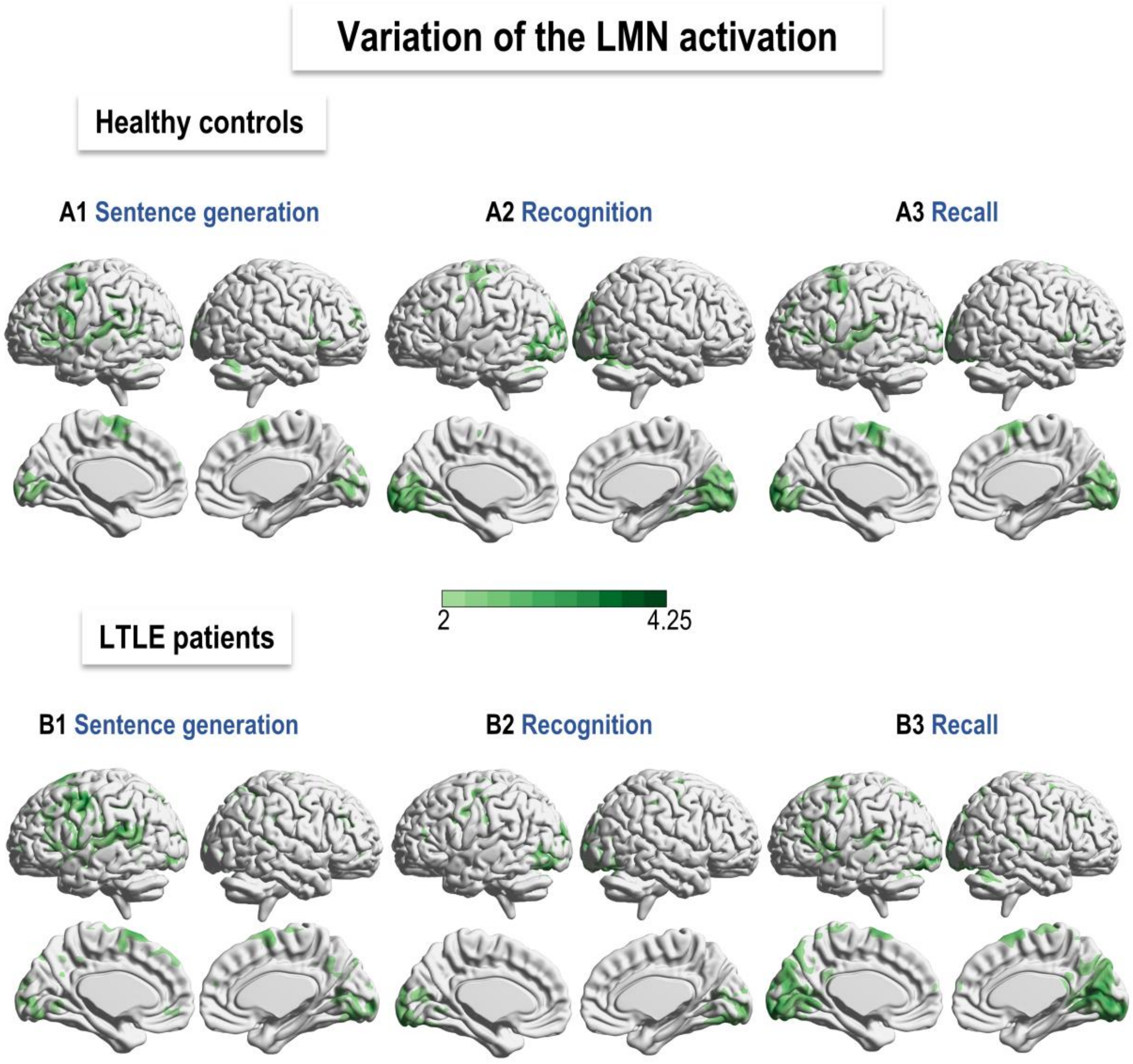

Figure S1. Illustration of activation variation in HC (**A**) and LTLE (**B**) for each GE2REC task. The standard deviation (SD) of task activation across participants within a group is projected onto a 3D anatomical template for sentence generation (**A1, B1**), recognition (**A2, B2**), and recall (**A3, B3**). The color scale indicates the SD value.

Table S2. Standard deviation (SD) for each AAL region for GE2REC tasks in LTLE and HC groups. Regions with SD higher than 2SD are marked in red. The highest variation was found in occipital regions for LTLE during recall and in occipital regions in HC during recognition. **Abbreviations**: GE = sentence generation with implicit encoding; RECO = recognition; RA = recall; LTLE = Left temporal lobe epilepsy; HC = Healthy controls.

Lobe	AAL region	LTLE GE	LTLE RECO	LTLE RA	HC GE	HC RECO	HC RA
**Frontal**	Frontal_Inf_Oper_L	1.83	1.58	1.80	1.99	1.53	1.62
	Frontal_Inf_Oper_R	1.46	1.49	1.39	1.62	1.40	1.55
	Frontal_Inf_Orb_2_L	1.57	1.35	1.55	1.70	1.34	1.42
	Frontal_Inf_Orb_2_R	1.28	1.13	1.18	1.58	1.32	1.53
	Frontal_Inf_Tri_L	1.75	1.56	1.91	1.78	1.54	1.79
	Frontal_Inf_Tri_R	1.45	1.51	1.48	1.48	1.42	1.67
	Frontal_Med_Orb_L	1.51	0.95	1.13	1.13	1.12	1.03
	Frontal_Med_Orb_R	1.43	1.03	1.23	1.16	1.05	1.07
	Frontal_Mid_2_L	1.72	1.32	1.61	1.58	1.37	1.64
	Frontal_Mid_2_R	1.57	1.44	1.47	1.45	1.39	1.44
	Frontal_Sup_2_L	1.49	1.29	1.49	1.43	1.30	1.40
	Frontal_Sup_2_R	1.39	1.34	1.36	1.41	1.27	1.31
	Frontal_Sup_Medial_L	1.59	1.22	1.46	1.41	1.08	1.29
	Frontal_Sup_Medial_R	1.45	1.25	1.36	1.42	1.09	1.26
	OFCant_L	1.05	0.82	0.82	0.82	0.84	0.76
	OFCant_R	0.84	0.73	0.77	0.68	0.92	0.74
	OFClat_L	1.04	1.01	0.89	0.95	0.76	0.76
	OFClat_R	0.84	0.89	0.78	0.96	0.90	0.72
	OFCmed_L	0.72	0.55	0.87	0.61	0.68	0.56
	OFCmed_R	0.57	0.54	0.52	0.58	0.69	0.55
	OFCpost_L	1.34	1.09	1.12	1.07	1.01	1.04
	OFCpost_R	1.18	1.02	1.08	1.22	1.13	1.04
	Olfactory_L	1.10	1.20	1.29	0.99	1.05	1.09
	Olfactory_R	0.98	1.07	1.28	1.00	1.05	1.05
	Paracentral_Lobule_L	1.48	0.96	1.52	1.29	1.26	1.22
	Paracentral_Lobule_R	1.54	0.99	1.60	1.27	1.14	1.34
	Precentral_L	1.49	1.31	1.50	1.34	1.36	1.34
	Precentral_R	1.45	1.46	1.47	1.21	1.23	1.19
	Rectus_L	0.72	0.60	0.86	0.75	0.67	0.70
	Rectus_R	0.68	0.58	0.68	0.76	0.69	0.74
	Rolandic_Oper_L	1.75	1.24	1.46	1.36	1.24	1.43
	Rolandic_Oper_R	1.52	1.31	1.24	1.17	1.12	1.35
	Supp_Motor_Area_L	1.90	1.42	1.72	1.95	1.48	1.91
	Supp_Motor_Area_R	1.59	1.25	1.66	1.54	1.41	1.64
**Insula and Cingulate**	Cingulate_Ant_L	1.53	1.24	1.29	1.20	1.10	1.24
	Cingulate_Ant_R	1.40	1.28	1.21	1.20	1.13	1.11
	Cingulate_Mid_L	1.51	1.12	1.33	1.35	1.23	1.30
	Cingulate_Mid_R	1.47	1.19	1.30	1.38	1.25	1.30
	Cingulate_Post_L	1.52	1.24	1.72	1.37	1.05	1.26
	Cingulate_Post_R	1.59	1.27	1.69	1.33	1.01	1.23
	Insula_L	1.50	1.15	1.36	1.24	1.16	1.39

	Insula_R	1.55	1.28	1.32	1.29	1.14	1.37
**Temporal**	Amygdala_L	1.01	1.28	1.34	1.16	0.99	1.15
	Amygdala_R	1.16	1.12	0.99	1.12	0.97	0.91
	Fusiform_L	1.28	1.54	1.41	1.15	1.71	1.27
	Fusiform_R	1.30	1.41	1.34	1.10	1.73	1.16
	Heschl_L	1.38	1.02	1.39	1.14	1.12	1.42
	Heschl_R	1.35	1.17	1.30	1.11	1.00	1.43
	Hippocampus_L	1.11	1.17	1.13	1.03	0.97	1.13
	Hippocampus_R	1.29	1.09	1.11	1.02	0.97	1.01
	ParaHippocampal_L	0.98	1.23	1.15	1.04	1.01	0.97
	ParaHippocampal_R	1.17	1.12	1.12	1.08	1.09	0.96
	Temporal_Inf_L	1.05	1.04	0.99	0.98	1.09	1.06
	Temporal_Inf_R	1.05	1.01	0.98	0.91	1.11	0.94
	Temporal_Mid_L	1.53	1.15	1.51	1.65	1.19	1.52
	Temporal_Mid_R	1.36	1.14	1.48	1.30	1.30	1.30
	Temporal_Pole_Mid_L	0.85	0.64	0.83	1.07	0.66	0.83
	Temporal_Pole_Mid_R	0.99	0.75	0.95	0.96	0.74	0.84
	Temporal_Pole_Sup_L	1.29	0.91	1.47	1.57	0.93	1.20
	Temporal_Pole_Sup_R	1.29	0.92	1.38	1.36	1.03	1.35
	Temporal_Sup_L	1.85	1.23	1.64	1.71	1.16	1.80
	Temporal_Sup_R	1.49	1.08	1.65	1.44	1.14	1.60
**Parietal**	Angular_L	1.78	1.20	1.80	1.54	1.25	1.38
	Angular_R	1.57	1.30	1.40	1.39	1.42	1.17
	Parietal_Inf_L	1.81	1.55	1.67	1.58	1.47	1.48
	Parietal_Inf_R	1.87	1.37	1.47	1.31	1.45	1.29
	Parietal_Sup_L	1.54	1.32	1.51	1.19	1.53	1.20
	Parietal_Sup_R	1.57	1.19	1.48	1.15	1.36	1.04
	Postcentral_L	1.74	1.49	1.54	1.33	1.57	1.26
	Postcentral_R	1.43	1.27	1.46	1.11	1.05	1.11
	Precuneus_L	1.54	1.11	1.74	1.27	1.23	1.27
	Precuneus_R	1.62	1.14	1.68	1.30	1.25	1.30
	SupraMarginal_L	2.01	1.54	1.64	1.98	1.63	1.75
	SupraMarginal_R	1.77	1.39	1.67	1.31	1.31	1.38
**Occipital**	Calcarine_L	1.68	1.90	2.26	1.77	2.09	1.92
	Calcarine_R	1.92	1.89	2.39	1.74	2.10	1.86
	Cuneus_L	1.51	1.44	2.00	1.52	1.56	1.53
	Cuneus_R	1.79	1.61	2.29	1.70	1.82	1.66
	Lingual_L	1.69	1.81	2.10	1.57	2.05	1.68
	Lingual_R	1.82	1.83	2.23	1.52	2.08	1.79
	Occipital_Inf_L	1.83	2.14	2.07	1.51	2.25	1.90
	Occipital_Inf_R	1.91	2.01	2.15	1.37	2.14	1.57
	Occipital_Mid_L	1.79	1.78	1.78	1.45	2.13	1.66
	Occipital_Mid_R	1.86	1.80	1.83	1.51	2.04	1.49
	Occipital_Sup_L	1.69	1.61	1.74	1.41	1.81	1.59
	Occipital_Sup_R	1.83	1.88	2.00	1.65	2.13	1.49
**Grey matter nuclei**	Caudate_L	1.28	1.10	1.23	1.00	1.14	1.13
	Caudate_R	1.28	1.15	1.33	1.09	1.04	1.05
	Pallidum_L	1.32	1.10	1.29	1.03	1.04	1.05
	Pallidum_R	1.34	1.21	1.09	0.90	1.03	1.03

	Putamen_L	1.16	1.12	1.26	1.07	1.06	1.17
	Putamen_R	1.25	1.20	1.13	1.02	1.00	1.17
	Thalamus_L	1.14	1.11	1.29	0.87	1.01	0.88
	Thalamus_R	1.16	1.14	1.18	0.96	1.08	0.95
**Cerebellum**	Cerebelum_10_L	1.14	0.81	1.02	0.74	1.06	0.64
	Cerebelum_10_R	1.02	0.82	0.94	0.80	1.16	0.74
	Cerebelum_3_L	1.00	1.18	1.36	0.96	1.12	1.00
	Cerebelum_3_R	1.22	1.16	1.34	0.94	1.20	1.07
	Cerebelum_4_5_L	1.30	1.21	1.43	1.13	1.25	1.07
	Cerebelum_4_5_R	1.35	1.45	1.41	1.06	1.50	1.18
	Cerebelum_6_L	1.52	1.59	1.75	1.45	1.89	1.54
	Cerebelum_6_R	1.76	1.59	1.88	1.52	1.99	1.51
	Cerebelum_7b_L	1.11	1.08	0.99	0.83	1.07	0.77
	Cerebelum_7b_R	1.27	1.00	1.14	1.18	0.93	0.91
	Cerebelum_8_L	1.16	1.04	1.01	0.86	0.95	0.89
	Cerebelum_8_R	1.28	1.17	1.11	1.04	1.04	0.89
	Cerebelum_9_L	1.08	0.92	1.08	0.89	0.89	0.80
	Cerebelum_9_R	1.10	0.98	1.03	0.84	0.91	0.74
	Cerebelum_Crus1_L	1.27	1.34	1.52	1.23	1.85	1.43
	Cerebelum_Crus1_R	1.51	1.27	1.72	1.38	1.60	1.36
	Cerebelum_Crus2_L	1.11	1.11	1.22	0.99	1.20	1.05
	Cerebelum_Crus2_R	1.31	0.94	1.33	1.12	0.96	0.99
	Vermis_1_2	0.97	1.35	1.32	0.96	1.16	0.97
	Vermis_10	1.09	1.22	1.60	1.12	1.16	1.00
	Vermis_3	1.15	1.24	1.47	1.19	1.02	0.97
	Vermis_4_5	1.29	1.36	1.52	1.31	1.21	1.22
	Vermis_6	1.57	1.29	1.75	1.34	1.68	1.37
	Vermis_7	1.40	1.15	1.82	1.19	1.60	1.28
	Vermis_8	1.11	1.40	1.50	1.20	1.28	1.10
	Vermis_9	1.08	1.23	1.49	1.19	1.31	1.03

Table S3. Activated regions for the contrast GE (generation with implicit encoding) vs. baseline. For each peak, the number of voxels in the cluster (k), T value, x, y, z coordinates (in mm) and region label (AAL atlas, Tzourio-Mazoyer et al., 2002) are presented. Activations were obtained at $T_{GE} > 6.89$ ($p < 0.05$, FWE). T values marked with * were obtained at $T_{GE} > 3.65$ ($p < 0.001$, uncorrected). Regions in bold represent cluster peaks.

Contrast	k	T	x	y	z	AAL
**GE vs. baseline**	**69**	**9.99**	**42**	**-60**	**-30**	**Cerebelum_Crus1_R**
		7.97	33	-60	-27	Cerebelum_6_R
		7.74	18	-87	-6	Lingual_R
		6.67*	27	-93	12	Occipital_Mid_R
		7.65	21	-96	15	Occipital_Sup_R
		5.14*	24	-81	-15	Fusiform_R
	**52**	**9.55**	**21**	**-96**	**9**	**Cuneus_R**
		8.64	15	-93	0	Calcarine_R
		7.69	21	-93	6	Occipital_Sup_R
	**34**	**9.36**	**-21**	**-93**	**3**	**Occipital_Mid_L**

		7.71	-15	-90	-12	Lingual_L
	**40**	**9.32**	**-3**	**12**	**60**	**Supp_Mot_Area_L**
		6.76*	-54	-39	12	Temporal_Sup_L
	**42**	**9.28**	**60**	**-15**	**-9**	**Temporal_Sup_R**
		7.49	60	-12	-12	Temporal_Mid_R
	**22**	**8.58**	**-54**	**18**	**18**	**Frontal_Inf_Tri_L**
		8.24	-54	15	18	Frontal_Inf_Oper_L
		6.56*	-42	24	-6	Frontal_Inf_Orb_L
	**8**	**8.28**	**-60**	**-18**	**-3**	**Temporal_Mid_L**
	**8**	**7.85**	**-39**	**15**	**21**	**Frontal_Inf_Tri_L**
	**5**	**7.46**	**-57**	**-39**	**3**	**Temporal_Mid_L**
	**7**	**7.42**	**-45**	**-60**	**-27**	**Cerebelum_Crus1_L**
	68	5.9*	-15	-24	-18	Parahippocampal_L
		5.19*	-18	-24	-9	Hippocampus_L

Table S4. Activated regions for the contrast RECO (recognition) vs. baseline. For each peak, the number of voxels in the cluster (k), T value, x, y, z coordinates (in mm) and region label (AAL atlas, Tzourio-Mazoyer et al., 2002) and are presented. Activations were obtained at $T_{RECO} > 7.03$ ($p < 0.05$, FWE). T values marked with * were obtained at $T_{RECO} > 3.65$ ($p < 0.001$, uncorrected). Regions in bold represent cluster peaks.

**Contrast**	**k**	**T**	**x**	**y**	**z**	**AAL**
**RECO vs. baseline**	**828**	**20.26**	**33**	**-45**	**-18**	**Fusiform_R**
		15.02	36	-81	-12	Occipital_Inf_R
		14.05	21	-93	0	Calcarine_R
		16.4	30	-48	-21	Cerebelum_6_R
		8.68	45	-57	-15	Temporal_Inf_R
		8.08	24	-81	-9	Lingual_R
	**845**	**17.50**	**-30**	**-87**	**6**	**Occipital_Mid_L**
		14.50	-15	-90	-9	Occipital_Mid_L
		12.51	-30	-81	-6	Occipital_Inf_L
		11.44	-36	-51	-21	Fusiform_L
		11.17	-42	-57	-24	Cerebelum_6_L
		10.39	-42	-48	-15	Temporal_Inf_L
		7.68	-30	-81	-15	Lingual_L
	**155**	**11.88**	**3**	**15**	**45**	**Supp_Motor_Area_L**
		8.15	-3	6	57	Supp_Motor_Area_L
		7.81	-9	-3	54	Supp_Motor_Area_R
		8.5	-3	21	42	Frontal_Sup_Medial_L
		8.27	-6	12	42	Cingulate_Mid_L
		8.68	6	15	42	Cingulate_Mid_R
	**15**	**10.20**	**-21**	**-27**	**-3**	**Thalamus_L**
		7.13	-18	-27	3	Thalamus_L
		7.23	-21	-27	-6	Hippocampus_L
	**176**	**9.54**	**-27**	**-51**	**39**	**Parietal_Inf_L**

	9.52	-30	-9	60	Precentral_L
	8.50	-36	-36	39	Postcentral_L
	7.26	-27	-9	57	Frontal_Sup_2_L
**11**	**8.50**	**-48**	**3**	**27**	**Precentral_L**
**8**	**8.23**	**0**	**0**	**72**	**Supp_Motor_Area_L**
**17**	**7.64**	**-24**	**-6**	**6**	**Putamen_L**
**6**	**7.55**	**-30**	**21**	**3**	**Insula_L**
**6**	**7.52**	**-45**	**18**	**21**	**Frontal_Inf_Tri_L**
**5**	**7.45**	**30**	**21**	**0**	**Putamen_R**
**84**	**6.55***	**33**	**-3**	**-21**	**Amygdala_R**
	5.71*	30	15	-21	Insula_R
	4.95*	24	-6	-21	Hippocampus_R
**86**	**5.31***	**30**	**-54**	**48**	**Parietal_Inf_R**

Table S5. Activated regions for the contrast RA (sentence recall) vs. baseline. For each peak, the number of voxels in the cluster (k), T value, x, y, z coordinates (in mm) and region label (AAL atlas, Tzourio-Mazoyer et al., 2002) and are presented. Activations were obtained at $T_{RA} > 6.85$ ($p < 0.05$, FWE). T values marked with * were obtained at $T_{RA} > 3.65$ ($p < 0.001$, uncorrected). Regions in bold represent cluster peaks.

Contrast	k	T	x	y	z	AAL
**RA vs. baseline**	**113**	**10.51**	**3**	**30**	**42**	**Frontal_Sup_Medial_L**
		7.39	-3	15	51	Supp_Motor_Area_L
		10.4	6	27	42	Frontal_Sup_Medial_R
		9.24	2	24	4	Supp_Motor_Area_R
		4.85*	-27	-33	-3	Hippocampus_L
	**102**	**10.50**	**-57**	**-21**	**0**	**Temporal_Mid_L**
		7.76	-54	-36	9	Temporal_Sup_L
	**64**	**9.14**	**60**	**-12**	**0**	**Temporal_Sup_R**
	**85**	**4.68***	**-30**	**-63**	**42**	**Parietal_Inf_L**
	**43**	**8.85**	**30**	**-90**	**12**	**Occipital_Mid_R**
		8.15	27	-90	12	Occipital_Sup_R
	**23**	**8.57**	**-21**	**-96**	**3**	**Occipital_Mid_L**
	**34**	**8.32**	**-30**	**27**	**-3**	**Insula_L**
		7.40	-42	21	-9	Frontal_Inf_Orb_2_L
	**26**	**8.19**	**-48**	**18**	**24**	**Frontal_Inf_Tri_L**
		7.64	-51	15	21	Frontal_Inf_Oper_L
	**5**	**7.96**	**-36**	**6**	**51**	**Frontal_Mid_2_L**

Table S6. Percentage of the number of activated voxels in our regions of interest for language and memory included in the LMN. We generated maps for terms "language" and "memory" which yielded 1101 and 2744 studies respectively in the Neurosynth database. Those maps were binarized and added up. GE2REC LMN maps for HC and LTLE were obtained using second-level group analyses for each group for all three tasks. These maps were binarized (using as threshold $p < 0.001$, uncorrected and $k > 5$) and added up. A less permissive threshold ($p < 0.001$, uncorrected and $k > 5$) was used to binarize GE2REC activation given the limited number of participants compared to the number of meta-analyses and participants in Neurosynth. Each resulting image (Neurosynth LMN, LTLE LMN, and HC LMN) was projected on the AAL atlas. The percentage of activated voxels was calculated for each AAL region by dividing the number of activated voxels by the total number of voxels in a specific region. Regions including more than 30-50% activated voxels are yellow, 50-70% blue, and above 70% green. See also Figure 2. **Abbreviations:** HC = Healthy controls; LTLE = Left temporal lobe epilepsy.

Lobe	region	Neurosynth	GE2REC HC	GE2REC LTLE
**Frontal**	Frontal_Inf_Oper_L	81.41	84.78	87.76
	Frontal_Inf_Oper_R	8.36	38.10	21.02
	Frontal_Inf_Orb_2_L	69.29	60.81	53.93
	Frontal_Inf_Orb_2_R	2.97	29.63	18.31
	Frontal_Inf_Tri_L	77.50	69.67	74.22
	Frontal_Inf_Tri_R	25.10	18.78	37.94
	Frontal_Med_Orb_L	0.28	0.00	0.00
	Frontal_Med_Orb_R	0.00	0.00	0.00
	Frontal_Mid_2_L	36.15	14.66	16.17
	Frontal_Mid_2_R	23.21	8.48	3.29
	Frontal_Sup_2_L	9.30	14.28	13.87
	Frontal_Sup_2_R	9.19	2.30	0.20
	Frontal_Sup_Medial_L	15.74	16.88	11.90
	Frontal_Sup_Medial_R	1.78	7.45	6.94
	OFCant_L	2.26	0.00	0.90
	OFCant_R	0.46	0.31	0.00
	OFClat_L	61.93	0.00	0.00
	OFClat_R	0.00	0.00	0.00
	OFCmed_L	0.18	0.36	0.00
	OFCmed_R	0.00	0.32	0.00
	OFCpost_L	7.94	19.58	22.40
	OFCpost_R	0.53	13.19	9.27
	Olfactory_L	0.00	14.64	0.00
	Olfactory_R	0.00	2.42	6.92
	Paracentral_Lobule_L	0.00	0.00	0.96
	Paracentral_Lobule_R	0.00	0.00	0.00
	Precentral_L	32.90	58.14	59.61
	Precentral_R	3.43	18.60	0.59
	Rectus_L	0.23	0.00	0.00
	Rectus_R	0.00	2.15	0.00
	Rolandic_Oper_L	4.65	22.42	18.08
	Rolandic_Oper_R	1.35	2.10	6.69
	Supp_Motor_Area_L	28.78	67.68	67.91

	Supp_Motor_Area_R	1.56	47.20	32.60
**Insula and Cingulate**	Cingulate_Ant_L	0.07	30.64	6.93
	Cingulate_Ant_R	0.00	22.85	3.58
	Cingulate_Mid_L	6.03	23.13	17.52
	Cingulate_Mid_R	3.72	25.51	17.34
	Cingulate_Post_L	54.00	7.13	0.00
	Cingulate_Post_R	9.25	3.88	0.00
	Insula_L	9.47	54.47	35.90
	Insula_R	3.39	38.59	19.60
**Temporal**	Amygdala_L	0.00	67.73	0.00
	Amygdala_R	0.00	41.53	32.66
	Fusiform_L	35.19	66.06	58.66
	Fusiform_R	17.24	60.72	56.16
	Heschl_L	15.56	27.56	34.22
	Heschl_R	3.61	0.80	30.92
	Hippocampus_L	84.23	56.55	12.77
	Hippocampus_R	83.62	35.41	14.69
	ParaHippocampal_L	65.54	19.84	4.29
	ParaHippocampal_R	61.66	15.55	7.86
	Temporal_Inf_L	19.03	13.16	10.69
	Temporal_Inf_R	3.65	13.24	11.02
	Temporal_Mid_L	55.56	35.63	28.45
	Temporal_Mid_R	12.25	21.46	9.53
	Temporal_Pole_Mid_L	8.74	2.65	0.00
	Temporal_Pole_Mid_R	11.71	6.23	1.43
	Temporal_Pole_Sup_L	31.05	34.55	19.92
	Temporal_Pole_Sup_R	16.67	22.42	11.43
	Temporal_Sup_L	55.57	67.20	56.18
	Temporal_Sup_R	33.78	35.12	42.02
**Parietal**	Angular_L	44.93	9.38	11.17
	Angular_R	20.66	13.24	4.85
	Parietal_Inf_L	36.09	52.19	53.09
	Parietal_Inf_R	24.01	7.81	7.43
	Parietal_Sup_L	18.74	37.87	21.65
	Parietal_Sup_R	4.32	13.46	2.75
	Postcentral_L	6.27	50.00	39.11
	Postcentral_R	1.44	15.38	0.92
	Precuneus_L	20.80	2.55	1.16
	Precuneus_R	22.11	2.17	0.25
	SupraMarginal_L	5.41	12.10	15.84
	SupraMarginal_R	3.50	3.09	0.30
**Occipital**	Calcarine_L	7.48	66.08	27.77
	Calcarine_R	4.51	77.92	35.36
	Cuneus_L	7.01	17.37	0.07
	Cuneus_R	3.51	21.70	8.92
	Lingual_L	5.68	80.33	38.81
	Lingual_R	7.91	80.74	32.57
	Occipital_Inf_L	16.58	71.52	62.17
	Occipital_Inf_R	1.01	42.37	37.51

	Occipital_Mid_L	14.65	50.49	51.74
	Occipital_Mid_R	8.67	41.85	33.41
	Occipital_Sup_L	2.42	44.07	27.38
	Occipital_Sup_R	4.74	48.48	16.49
**Grey matter nuclei**	Caudate_L	3.12	19.02	8.00
	Caudate_R	0.80	59.26	6.34
	Pallidum_L	1.37	76.79	79.52
	Pallidum_R	0.00	41.43	42.86
	Putamen_L	0.50	82.06	69.38
	Putamen_R	0.75	32.80	20.49
	Thalamus_L	0.91	79.64	52.55
	Thalamus_R	0.76	44.75	8.42
**Cerebellum**	Cerebelum_10_L	0.00	0.00	0.00
	Cerebelum_10_R	0.00	0.00	0.00
	Cerebelum_3_L	4.41	1.47	0.00
	Cerebelum_3_R	5.80	1.45	0.00
	Cerebelum_4_5_L	4.62	34.22	15.73
	Cerebelum_4_5_R	11.85	73.05	32.06
	Cerebelum_6_L	0.71	79.99	58.50
	Cerebelum_6_R	6.96	84.51	85.13
	Cerebelum_7b_L	0.00	4.27	0.00
	Cerebelum_7b_R	4.31	0.00	0.00
	Cerebelum_8_L	0.00	6.68	0.00
	Cerebelum_8_R	2.12	3.16	1.60
	Cerebelum_9_L	0.00	1.73	0.00
	Cerebelum_9_R	0.12	3.83	0.00
	Cerebelum_Crus1_L	2.07	27.24	25.12
	Cerebelum_Crus1_R	9.03	30.10	29.53
	Cerebelum_Crus2_L	0.05	5.86	2.27
	Cerebelum_Crus2_R	2.74	4.96	3.12
	Vermis_1_2	0.00	0.00	0.00
	Vermis_10	0.00	0.00	0.00
	Vermis_3	1.32	17.11	18.42
	Vermis_4_5	3.61	55.79	22.26
	Vermis_6	5.12	93.26	29.38
	Vermis_7	33.51	90.72	62.37
	Vermis_8	0.82	66.26	0.41
	Vermis_9	0.00	47.13	0.00

Since data did not meet the criteria for ANOVA (normality W = 0.879, $p < .001$), we performed Kruskal–Wallis tests for each lobe. The results showed that there was no significant difference in the general coverage of the frontal ($\chi2(2) = 0.682$, $p = .711$), temporal ($\chi2\ (2) = 3.07$, $p = .216$), and parietal lobe ($\chi2(2) = 1.94$, $p = .379$) nor insula and cingulum ($\chi2(2) = 5.87$, $p = .05$). Although by comparing the percentages directly, we can see that the mesial temporal regions were less activated than expected, especially in the LTLE group. Significant differences between the general lobe coverage were found for grey matter nuclei ($\chi2(2) = 16.2$, $p < .001$; DSCF[1] post-hoc showed higher coverage using GE2REC both in HC (W = 4.75, p = .002) and LTLE (W = 4.75, $p = .002$) than based on Neurosynth maps, while there was no difference between HC and LTLE (W = -1.93, $p = .359$)), the occipital lobe ($\chi2(2) = 22.7$, $p < .001$, higher coverage using GE2REC both in HC (W = 5.88, $p < .001$) and LTLE (W = 4.65, $p = .003$) than based on Neurosynth maps, while the coverage was greater in HC than LTLE (W = -3.59, $p = .03$)) and the cerebellum ($\chi2\ (2) = 7.57$, $p = .023$, , higher coverage using GE2REC in HC (W = 3.756, $p = .022$) than based on Neurosynth maps, while there was no difference between Neurosynth and LTLE (W = 0.45, $p = .945$), nor between HC and LTLE (W = -2.845, $p = .109$)).

[1] Dwass-Steel-Critchlow-Flinger pairwise comparisons

Table S7. Lateralization indices for GE2REC tasks in LTLE and HC. Values in bold indicate bilateral (-0.2 < LI < 0.2) and those in red right hemispheric predominance (Seghier, 2019). LIs in LTLE marked with * are significantly different (p < .05) compared to HC (Crawford & Garthwaite, 2002). The number of participants in each group showing left, bilateral and right predominance for frontal, temporal and hippocampus for each task is presented in blue. Levene's test results for the equality of variance and Mann-Whitney U for testing differences between LTLE and HC for each lobe/structure are also presented. **Abbreviations:** N = participant number; LTLE = Left temporal lobe epilepsy; HC = Healthy controls; L = left-lateralized; B = bilateral; R = right lateralized; M = Mean; SD = standard deviation; Var diff = value of Levene's test for the equality of variances with corresponding p value; Diff = Values of Mann-Whitney U for testing differences between LTLE and HC with corresponding p value.

		Sentence generation with encoding			Recognition of items			Recall		
	**N**	**Frontal**	**Temporal**	**Hippocampus**	**Frontal**	**Temporal**	**Hippocampus**	**Frontal**	**Temporal**	**Hippocampus**
**LTLE**	P1	0.71	0.66	0.73	**-0.086***	0.41	**-0.39**	0.59	0.64	**-0.38**
	P2	0.71	0.53	0.38	**0.081***	**0.0034**	**-0.63**	0.7	0.46	0.69
	P3	0.82	**0.14**	0.28	**-0.37***	0.34	0.26	0.91	0.35	0.53
	P4	0.87	0.64	**0.14**	**-0.4***	**0.026**	**-0.55**	0.53	0.37	0.84
	P5	0.83	0.35	**-0.73**	**0.033***	**0.072**	0.49	0.56	0.24	**-0.31**
	P6	**-0.042***	0.27	**-0.73**	0.66	**-0.24**	**0.13**	**0.079**	**0.2**	0.96
	P7	0.86	0.38	0.81	0.52	**-0.29**	**-0.8**	0.43	0.32	**-0.38**
	P8	**-0.58***	**0.12**	**-0.57**	0.63	0.25	**-0.13**	**-0.81***	**-0.71***	**-0.15**
	P9	0.56	**0.14**	**0.1**	0.53	**-0.45***	0.25	**-0.14***	0.21	0.48
	P10	0.94	0.61	0.97	0.62	0.52	0.31	0.7	0.31	0.39
	P11	0.83	0.55	**0.059**	0.84	0.4	**0.11**	0.85	0.71	**0.11**
	P12	0.53	0.75	**-0.91**	0.67	**-0.35**	**-0.58**	**0.036**	0.52	0.86
	P13	**-0.52***	**-0.2***	**-0.55**	0.57	0.29	**-0.44**	**-0.42***	0.26	**-0.82**
	P14	0.76	0.63	0.75	0.58	**-0.51***	**-0.26**	0.46	0.55	0.64
	P15	0.8	0.65	0.44	0.56	**-0.19**	**-0.41**	0.84	0.75	0.81
	P16	0.43	0.74	**-0.74**	0.71	**0.15**	**-0.25**	0.69	0.4	**0.017**
	P17	0.71	0.81	0.77	0.78	**-0.34**	0.79	0.85	0.83	0.77
	P18	0.82	0.48	**-0.84**	0.57	**-0.2**	**-0.64**	0.73	0.5	**-0.8**
**HC**	HC1	0.65	0.68	0.94	0.52	**-0.12**	**0.17**	0.49	0.75	**0.11**
	HC2	0.79	0.58	**-0.29**	0.65	**-0.41**	0.39	0.62	**-0.12**	**-0.12**
	HC3	0.83	0.53	**-0.085**	0.43	**-0.14**	**-0.51**	0.6	0.56	**-0.017**

	HC4	**0.00**	**-0.077**	0.3	0.33	0.34	**-0.049**	**-0.12**	**0.19**	**-0.81**
	HC5	0.54	**0.031**	0.33	0.58	**0.0071**	0.77	0.49	**-0.34**	0.68
	HC6	0.70	0.58	0.56	0.73	**0.16**	0.53	0.82	0.71	0.83
	HC7	**0.12**	**0.2**	0.35	0.4	0.4	0.29	0.81	0.71	0.75
	HC8	0.70	0.72	**0.11**	0.38	0.49	0.24	0.67	0.63	**-0.24**
	HC9	0.76	0.68	0.67	0.7	**0.078**	**-0.057**	0.66	0.41	**0.1**
	HC10	0.50	**-0.039**	0.46	0.55	**-0.17**	0.027	0.57	0.38	**-0.87**
	HC11	0.72	0.58	**-0.49**	0.68	0.29	0.29	0.74	0.22	**-0.7**
	HC12	0.74	0.56	0.54	0.68	**-0.1**	0.51	0.42	**0.1**	**0.022**
	HC13	0.58	0.56	0.36	0.69	**0.068**	**-0.089**	0.46	**0.16**	0.63
	HC14	0.85	0.51	0.23	0.77	0.27	**-0.089**	0.72	**0.18**	0.35
	HC15	0.79	**0.015**	0.57	0.54	**0.16**	**-0.5**	0.51	**-0.21**	**-0.69**
	HC16	0.84	0.81	0.69	0.56	0.58	**-0.41**	0.77	0.76	**-0.9**
	HC17	0.67	0.62	0.63	0.75	**-0.29**	**-0.3**	0.58	0.68	0.85
	HC18	0.44	0.81	0.65	0.63	**0.15**	0.5	**0.061**	0.53	0.47
	HC19	0.53	0.57	0.59	0.64	0.23	**0.052**	0.57	0.54	0.57
**LTLE**	L	15 (83.3%)	14 (77.8%)	8 (44.4%)	13 (72.2%)	6 (33.3%)	5 (27.8%)	13 (72,2%)	16 (88.9%)	10 (55.6%)
	B	1 (5.6%)	4 (22.2%)	3 (16.7%)	3 (16.7%)	6 (33.3%)	3 (16.7%)	3 (16.7%)	1 (5.6%)	3 (16.7%)
	R	2 (11.1%)	0 (0%)	7 (38.9%)	2 (11.1%)	6 (33.3%)	10 (55.6%)	2 (11.1%)	1 (5.6%)	5 (27.8%)
**HC**	L	17 (89.5%)	14 (73.7%)	15 (78.9%)	19 (100%)	7 (36.8%)	8 (42.1%)	17 (89.5%)	12 (63.2%)	8 (42.1%)
	B	2 (10.5%)	5 (26.3%)	2 (10.5%)	0 (0%)	10 (52.6%)	7 (36.8%)	2 (10.5%)	5 (26.3%)	5 (26.3%)
	R	0 (0%)	0 (0 %)	2 (10.5%)	0 (0%)	2 (10.5%)	4 (21.1%)	0 (0%)	2 (105%)	6 (31.6%)
**M**	LTLE	0.56	0.46	0.02	0.42	-0.01	-0.15	0.42	0.38	0.24
	HC	0.62	0.47	0.37	0.59	0.11	0.09	0.55	0.36	0.05
**SD**	LTLE	0.462	0.273	0.664	0.385	0.326	0.45	0.11	0.332	0.139
	HC	0.231	0.289	0.358	0.131	0.265	0.367	0.05	0.339	0.14
**Var diff.**		3.98	0.04	12.69	16.45	1.85	1.356	7.36	1.05	0.003
p		0.054	0.842	<.001*	<.001*	0.183	0.252	0.01*	0.310	0.953
**Diff**		145	167	124	138	135	112	167	163	139
p		0.438	0.903	0.158	0.323	0.274	0.073	0.903	0.808	0.331

Table S8. Activation differences between healthy and patients during GE (generation with implicit encoding). For each peak, the number of voxels in the cluster (k), T value, x, y, z coordinates (in mm) and region label (AAL atlas, Tzourio-Mazoyer et al., 2002) are presented. All activations were obtained at T > 3.35 ($p < 0.001$, uncorrected). Regions in bold represent cluster peaks.

Contrast	k	T	x	y	z	AAL
**HC> LTLE**	**155**	**5.05**	**-15**	**-12**	**48**	**Cingulate_Mid_L**
		4.59	-6	12	51	Supp_Motor_Area_L
		4.43	12	12	57	Supp_Motor_Area_R
	**80**	**4.95**	**-21**	**-54**	**45**	**Parietal_Sup_L**
		3.51	-27	-51	48	Parietal_Inf_L
	**156**	**4.81**	**45**	**-18**	**45**	**Precentral_R**
		4.37	45	-36	48	Parietal_Inf_R
		4.11	42	-27	54	Postcentral_R
	**84**	**4.51**	**27**	**45**	**21**	**Frontal_Sup_2_R**
		4.12	33	36	24	Frontal_Mid_2_R
	**9**	**4.48**	**-9**	**-21**	**0**	**Thalamus_L**
	**29**	**4.47**	**48**	**0**	**30**	**Precentral_R**
	**50**	**4.32**	**54**	**12**	**3**	**Frontal_Inf_Oper_R**
	**27**	**4.25**	**6**	**6**	**0**	**Caudate_R**
	**10**	**4.12**	**-36**	**12**	**30**	**Frontal_Inf_Oper_L**
	**23**	**4.08**	**-48**	**9**	**0**	**Frontal_Inf_Oper_L**
	**20**	**4.06**	**-9**	**18**	**30**	**Cingulate_Ant_L**
	**5**	**4.04**	**6**	**-30**	**66**	**Paracentral_Lobule_R**
	**12**	**3.93**	**-15**	**12**	**-3**	**Putamen_L**
	**18**	**3.84**	**-48**	**-33**	**6**	**Temporal_Sup_L**
	**19**	**3.73**	**-45**	**0**	**45**	**Precentral_L**
		3.72	-36	-3	42	Precentral_L
	**7**	**3.71**	**-12**	**-42**	**51**	**Cingulate_Mid_L**
	**6**	**3.69**	**9**	**-24**	**51**	**Supp_Motor_Area_R**
	**25**	**3.65**	**24**	**-57**	**42**	**Angular_R**
		3.50	12	-66	42	Precuneus_R
	**6**	**3.57**	**-33**	**18**	**-3**	**Insula_L**
	**7**	**3.54**	**-45**	**-18**	**42**	**Postcentral_L**
**LTLE > HC**	No suprathreshold clusters					

Table S9. Activation differences between healthy and patients during RECO (recognition). For each peak, the number of voxels in the cluster (k), T value, x, y, z coordinates (in mm) and region label (AAL atlas, Tzourio-Mazoyer et al., 2002) are presented. All activations were obtained at T > 3.35 ($p < 0.001$, uncorrected). Regions in bold represent cluster peaks.

Contrast	k	T	x	y	z	AAL
**HC > LTLE**	**63**	**5.07**	**-33**	**-36**	**45**	**Postcentral_L**
		3.76	-27	-45	42	Parietal_Inf_L
	**70**	**4.58**	**27**	**-54**	**-12**	**Fusiform_R**

		3.77	21	-45	-15	Fusiform_R
		3.60	45	-48	0	Temporal_Mid_R
	**12**	**4.25**	**24**	**-84**	**-9**	**Lingual_R**
	**10**	**3.89**	**-45**	**-21**	**24**	**Rolandic_Oper_L**
	**12**	**3.89**	**27**	**-72**	**51**	**Parietal_Sup_R**
	**8**	**3.88**	**-33**	**-18**	**57**	**Precentral_L**
	**12**	**3.86**	**-33**	**-75**	**15**	**Occipital_Mid_L**
		3.61	-24	-84	15	Occipital_Mid_L
	**15**	**3.86**	**-24**	**-63**	**-9**	**Lingual_L**
		3.76	-27	-60	-9	Fusiform_L
	**8**	**3.69**	**-39**	**-75**	**-3**	**Occipital_Mid_L**
		3.63	-33	-84	-3	Occipital_Mid_L
	**5**	**3.67**	**0**	**3**	**30**	**Cingualate_Ant_L**
	**11**	**3.64**	**45**	**-54**	**-15**	**Temporal_Inf_R**
	**8**	**3.57**	**36**	**-75**	**0**	**Occipital_Mid_R**
**LTLE > HC**	No suprathreshold clusters					

Table S10. Activation differences between healthy and patients during RA (sentence recall). For each peak, the number of voxels in the cluster (k), T value, x, y, z coordinates (in mm) and region label (AAL atlas, Tzourio-Mazoyer et al., 2002) are presented. Activations were obtained at T > 3.35 ($p < 0.001$, uncorrected). Regions in bold represent cluster peaks.

Contrast	k	T	x	y	z	AAL
**HC > LTLE**	**19**	**4.40**	**51**	**15**	**3**	**Frontal_Inf_Oper_R**
	**13**	**4.08**	**39**	**12**	**-9**	**Insula_R**
	**21**	**3.82**	**0**	**15**	**51**	**Supp_Motor_Area_L**
		3.43	6	15	54	Supp_Motor_Area_R
	**11**	**3.73**	**36**	**24**	**0**	**Insula_R**
**LTLE > HC**	No suprathreshold clusters					

Table S11. Activated regions for the contrast GE (generation with implicit encoding) vs. baseline for P1 before surgery. For each peak, the number of voxels in the cluster (k), T value, x, y, z coordinates (in mm) and region label (AAL atlas, Tzourio-Mazoyer et al., 2002) are presented. Activations were obtained at T > 4.59 ($p < 0.05$, FWE). Values marked with * were obtained at T > 3.1 ($p < 0.001$, uncorrected). Regions in bold represent cluster peaks.

Contrast	k	T	x	y	z	AAL
**GE vs. baseline**	**172**	**6.47**	**-63**	**-6**	**-6**	**Temporal_Mid_L**
		6.40	-57	-30	0	Temporal_Mid_L
		5.73	-48	-33	0	Temporal_Mid_L
	**11**	**6.16**	**-9**	**-96**	**24**	**Cuneus_L**
		4.35*	-15	-102	12	Occipital_Sup_L
	**15**	**5.58**	**54**	**-27**	**0**	**Temporal_Sup_R**
	**15**	**5.51**	**21**	**-69**	**-48**	**Cerebelum_8_R**
	**114***	**4.96**	**-15**	**6**	**72**	**Frontal_Sup_2_L**
	**6**	**4.89**	**6**	**-81**	**-3**	**Lingual_R**

	4.71	12	-78	-9	Lingual_R
**114**	**4.71**	**-9**	**-63**	**-3**	**Lingual_L**
	4.50*	-30	27	-3	Insula_L
	4.22*	-30	21	-9	Insula_L
**20**	**3.66***	**-30**	**18**	**-18**	**Insula_L**
	4.40*	-57	-24	21	SupraMarginal_L
**55**	**4.23***	**-21**	**-78**	**-21**	**Cerebelum_6_L**
	3.48*	-36	-72	-24	Cerebelum_Crus1_L
**47**	**4.20***	**33**	**24**	**6**	**Insula_R**
**36**	**4.01***	**-51**	**24**	**24**	**Frontal_Inf_Tri_L**
	3.77*	-42	24	21	Frontal_Inf_Tri_L
**51**	**3.99***	**-42**	**-60**	**-33**	**Cerebelum_Crus1_L**
	3.77*	-48	-63	-39	Cerebelum_Crus1_L
**8**	**3.62***	**-6**	**42**	**12**	**Cingulate_Ant_L**
**17**	**3.61***	**60**	**0**	**-12**	**Temporal_Sup_R**
**18**	**3.56***	**-42**	**-42**	**-15**	**Temporal_Inf_L**
**6**	**3.47***	**21**	**-27**	**24**	**Caudate_R**
**5**	**3.45***	**-33**	**42**	**15**	**Frontal_Mid_2_L**
**11**	**3.39***	**-21**	**51**	**27**	**Frontal_Sup_2_L**
**5**	**3.34***	**-24**	**-36**	**0**	**Hippocampus_L**
**6**	**3.32***	**-15**	**-93**	**-12**	**Lingual_L**
**7**	**3.32***	**45**	**-63**	**-30**	**Cerebelum_Crus1_R**

Table S12. Activation differences between P1 and HC during GE (generation with implicit encoding) before surgery. For each peak, the number of voxels in the cluster (k), T value, x, y, z coordinates (in mm) and region label (AAL atlas, Tzourio-Mazoyer et al., 2002) and are presented. Activations were obtained at T > 3.61 (*p* < 0.001, uncorrected). Regions in bold represent cluster peaks.

Contrast	k	T	x	y	z	AAL
**HC > P1**	**56**	**5.89**	**18**	**-54**	**39**	**Precuneus_R**
**P1 > HC**	**9**	**7.86**	**24**	**-39**	**30**	**Cingulate_Mid_R**
		4.51	60	-24	36	SupraMarginal_R
	**9**	**4.73**	**-27**	**-39**	**-3**	**Hippocampus_L**
	**9**	**4.63**	**33**	**-54**	**12**	**Calcarine_R**
	**5**	**4.51**	**60**	**-24**	**36**	**SupraMarginal_R**
	**5**	**4.06**	**-39**	**-42**	**-9**	**Temporal_Inf_L**

Table S13. Activated regions for the contrast GE (generation with implicit encoding) vs. baseline in P1 after surgery. For each peak, the number of voxels in the cluster (k), T value, x, y, z coordinates (in mm) and region label (AAL atlas, Tzourio-Mazoyer et al., 2002) and are presented. Activations were obtained at T > 4.58 (*p* < 0.05, FWE). Values marked with * were obtained at T > 3.1 (*p* < 0.001, uncorrected). Regions in bold represent cluster peaks.

Contrast	k	T	x	y	z	AAL
**GE**	**79**	**6.81**	**-9**	**9**	**72**	**Supp_Motor_Area_L**
	**57**	**5.78**	**-54**	**6**	**0**	**Temporal_Pole_Sup_L**

**vs. baseline**		5.53	-51	6	9	Frontal_Inf_Oper_L
		4.10*	-33	15	9	Insula_L
	**57**	**5.77**	**-54**	**-30**	**-6**	**Temporal_Mid_L**
		5.26	-54	-21	-15	Temporal_Mid_L
	**23**	**4.48***	**-42**	**0**	**57**	**Precentral_L**
	**13**	**4.48***	**24**	**-69**	**-51**	**Cerebelum_8_R**
	**27**	**4.11***	**-21**	**45**	**27**	**Frontal_Sup_2_L**
	**33**	**4.02***	**-48**	**6**	**33**	**Precentral_L**
		3.29*	-33	3	33	Precentral_L
	**29**	**3.93***	**-42**	**-45**	**-24**	**Fusiform_L**
	**16**	**3.89***	**-15**	**-99**	**9**	**Occipital_Mid_L**
	**10**	**3.60***	**-18**	**-81**	**-21**	**Cerebelum_Crus1_L**
		3.32*	-27	-75	-21	Cerebelum_6_L
	**7**	**3.42***	**9**	**18**	**36**	**Cingulate_Mid_R**

Table S14. Activation differences between P1 and HC during GE (generation with implicit encoding) after surgery. For each peak, the number of voxels in the cluster (k), T value, x, y, z coordinates (in mm) and region label (AAL atlas, Tzourio-Mazoyer et al., 2002) are presented. All activations were obtained at T > 3.61 ($p < 0.001$, uncorrected). Regions in bold represent cluster peaks.

Contrast	k	T	x	y	z	AAL
**HC > P1**	**14**	**5.88**	**12**	**-57**	**51**	**Precuneus_R**
	**18**	**5.08**	**-12**	**-63**	**45**	**Precuneus_L**
	**7**	**4.92**	**42**	**-48**	**12**	**Temporal_Mid_R**
	**8**	**4.76**	**-42**	**-39**	**9**	**Temporal_Sup_L**
	**22**	**4.47**	**-21**	**-48**	**48**	**Parietal_Sup_L**
	**6**	**4.43**	**6**	**-66**	**42**	**Precuneus_R**
	**17**	**4.33**	**57**	**-36**	**9**	**Temporal_Sup_R**
		4.33	63	-30	15	Temporal_Sup_R
	**7**	**4.19**	**-3**	**-12**	**6**	**Thalamus_L**
	**5**	**3.86**	**18**	**-45**	**60**	**Parietal_Sup_R**
**P1 > HC**	**25**	**5.53**	**-48**	**-33**	**-12**	**Temporal_Mid_L**
		4.42	-51	-24	-15	Temporal_Mid_L

Table S15. Activated regions for the contrast RECO (recognition) vs. baseline in P1 before surgery. For each peak, the number of voxels in the cluster (k), T value, x, y, z coordinates (in mm) and region label (AAL atlas, Tzourio-Mazoyer et al., 2002) are presented. Activations were obtained at T > 4.59 ($p < 0.05$, FWE). T values marked with * were obtained at T > 3.1 ($p < 0.001$, uncorrected). Regions in bold represent cluster peaks.

Contrast	k	T	x	y	z	AAL
**RECO vs. baseline**	**628**	**8.57**	**36**	**-87**	**6**	**Occipital_Mid_R**
		7.49	33	-72	-15	Fusiform_R
		6.77	21	-93	-9	Lingual_R
	**640**	**8.51**	**-33**	**-81**	**-18**	**Fusiform_L**
		8.48	-30	-54	-21	Cerebelum_6_L
		7.27	-45	-75	-12	Occipital_Inf_L

**184**	**7.79**	**42**	**-12**	**63**	**Precentral_R**
	6.21	18	-6	72	Frontal_Sup_2_R
	6.18	45	-24	60	Precentral_R
**93**	**6.45**	**-54**	**9**	**36**	**Precentral_L**
	4.88	-45	0	51	Precentral_L
**52**	**6.39**	**-30**	**-60**	**48**	**Parietal_Inf_L**
**83**	**5.43**	**-6**	**6**	**45**	**Supp_Motor_Area_L**
	5.41	6	9	45	Supp_Motor_Area_R
	5.02	-3	-3	54	Supp_Motor_Area_L
**274**	**6.39**	**-30**	**-60**	**48**	**Parietal_Inf_L**
	4.50*	-36	-51	39	Parietal_Inf_L
	4.18*	-39	-39	36	Parietal_Inf_L
**187**	**4.55***	**12**	**-66**	**6**	**Calcarine_R**
	4.40*	24	-63	3	Calcarine_R
	3.67*	6	-75	-6	Lingual_R
**40**	**4.54***	**39**	**3**	**24**	**Frontal_Inf_Oper_R**
**117**	**4.40***	**21**	**-30**	**-3**	**Hippocampus_R**
	3.81*	9	-21	3	Thalamus_R
	3.80*	12	-12	6	Thalamus_R
**272**	**4.29***	**-36**	**30**	**-12**	**Frontal_Inf_Orb_2_L**
	4.24*	-33	21	0	Insula_L
	4.05*	-30	18	9	Insula_L
**10**	**4.29***	**-57**	**-21**	**42**	**SupraMarginal_L**
**14**	**4.26***	**66**	**0**	**18**	**Postcentral_R**
**41**	**4.15***	**21**	**0**	**-18**	**Amygdala_R**
	3.37*	30	9	-27	Temporal_Pole_Sup_R
	3.18*	18	-6	-9	Hippocampus_R
**55**	**4.10***	**-15**	**6**	**-21**	**ParaHippocampal_L**
	3.63*	-18	-6	-21	Hippocampus_L
**12**	**4.07***	**-15**	**-3**	**75**	**Frontal_Sup_2_L**
**10**	**3.99***	**15**	**-75**	**-48**	**Cerebelum_8_R**
**27**	**3.75***	**39**	**15**	**6**	**Insula_R**
	3.42*	30	24	0	Insula_R
**16**	**3.74***	**-51**	**6**	**-3**	**Temporal_Pole_Sup_L**
**22**	**3.42***	**18**	**6**	**0**	**Pallidum_R**
	3.35*	27	0	3	Putamen_R
**8**	**3.35***	**3**	**30**	**30**	**Cingulate_Mid_R**

Table S16. Activation differences between P1 and HC during RECO (recognition) before surgery. For each peak, the number of voxels in the cluster (k), T value, x, y, z coordinates (in mm) and region label (AAL atlas, Tzourio-Mazoyer et al., 2002) are presented. All activations were obtained at T > 3.61 ($p < 0.001$, uncorrected). Regions in bold represent cluster peaks.

Contrast	k	T	x	y	z	AAL
**HC > P1**	**30**	**4.92**	**-21**	**-42**	**21**	**Cingulate_Post_L**
	**10**	**4.50**	**9**	**-48**	**-12**	**Cerebelum_4_5_R**
**P1 > HC**	**37**	**7.01**	**-18**	**-3**	**-24**	**ParaHippocampal_L**

	4.76	-12	-12	-21	Hippocampus_L
**39**	**6.70**	**12**	**-6**	**75**	**Supp_Motor_Area_R**
	4.53	24	-15	72	Precentral_R
	3.80	12	6	72	Supp_Motor_Area_R
**17**	**5.85**	**33**	**3**	**-24**	**Temporal_Pole_Sup_R**
**11**	**5.38**	**63**	**0**	**18**	**Postcentral_R**
**70**	**5.24**	**45**	**-12**	**51**	**Precentral_R**
	5.08	45	-24	54	Postcentral_R
**14**	**4.74**	**24**	**-36**	**0**	**Hippocampus_R**
**50**	**4.46**	**-9**	**-30**	**6**	**Thalamus_L**
	4.41	12	-24	3	Thalamus_R
	4.26	3	-30	12	Thalamus_R
**5**	**4.17**	**3**	**-6**	**18**	**Thalamus_R**
**5**	**3.76**	**39**	**12**	**6**	**Insula_R**

Table S17. Activated regions for the contrast RECO (recognition) vs. baseline in P1 after surgery. For each peak, the number of voxels in the cluster (k), T value, x, y, z coordinates (in mm) and region label (AAL atlas, Tzourio-Mazoyer et al., 2002) are presented. Activations were obtained at T > 4.58 ($p < 0.05$, FWE). T values marked with * were obtained at T > 3.1 ($p < 0.001$, uncorrected). Regions in bold represent cluster peaks.

Contrast	k	T	x	y	z	AAL
**RECO vs. baseline**	**158**	**6.16**	**-18**	**-90**	**3**	**Occipital_Sup_L**
		6.13	-27	-87	9	Occipital_Mid_L
		5.62	-24	-93	15	Occipital_Mid_L
	**107**	**6.05**	**27**	**-81**	**-9**	**Fusiform_R**
		5.94	21	-93	15	Occipital_Sup_R
		4.83	15	-96	3	Calcarine_R
	**70**	**5.65**	**-36**	**-78**	**-12**	**Occipital_Inf_L**
		4.99	-36	-60	-24	Cerebelum_6_L
		4.59	-30	-72	-9	Fusiform_L
	**5**	**4.80**	**-39**	**0**	**33**	**Precentral_L**
	**47**	**4.79**	**-30**	**-63**	**45**	**Parietal_Sup_L**
	**66**	**4.24***	**-3**	**-6**	**57**	**Supp_Motor_Area_L**
		3.33*	-3	3	48	Supp_Motor_Area_L
	**7**	**3.84***	**15**	**-90**	**33**	**Cuneus_R**
	**15**	**3.64***	**15**	**-6**	**69**	**Supp_Motor_Area_R**
	**10**	**3.59***	**-42**	**-18**	**45**	**Postcentral_L**
	**12**	**3.56***	**-39**	**-15**	**57**	**Precentral_L**

Table S18. Activation differences between P1 and HC during RECO (recognition) after surgery. For each peak, the number of voxels in the cluster (k), T value, x, y, z coordinates (in mm) and region label (AAL atlas, Tzourio-Mazoyer et al., 2002) are presented. All activations were obtained at T > 3.61 ($p < 0.001$, uncorrected). Regions in bold represent cluster peaks.

Contrast	k	T	x	y	z	AAL
**HC > P1**	**277**	**9.59**	**36**	**-45**	**15**	**Temporal_Mid_R**
		6.80	21	-42	24	Precuneus_R

		6.74	39	-39	21	Temporal_Sup_R
	**58**	**7.10**	**-24**	**-54**	**21**	**Cuneus_L**
		5.43	-15	-48	21	Cingulate_Post_L
	**20**	**6.05**	**-27**	**-6**	**-21**	**Hippocampus_L**
	**9**	**5.60**	**-42**	**-69**	**42**	**Angular_L**
	**8**	**5.44**	**-9**	**24**	**60**	**Supp_Motor_Area_L**
	**7**	**5.43**	**-54**	**-12**	**-6**	**Temporal_Sup_L**
	**10**	**5.26**	**27**	**-75**	**48**	**Parietal_Sup_R**
	**29**	**5.20**	**45**	**-9**	**-12**	**Temporal_Sup_R**
		4.31	60	-6	-21	Temporal_Mid_R
		4.04	54	-12	-18	Temporal_Mid_R
	**13**	**5.14**	**-42**	**-42**	**0**	**Temporal_Mid_L**
	**17**	**5.11**	**12**	**30**	**54**	**Frontal_Sup_Medial_R**
	**23**	**5.04**	**-60**	**-57**	**3**	**Temporal_Mid_L**
	**19**	**5.02**	**-42**	**-18**	**-15**	**Temporal_Inf_L**
	**20**	**5.01**	**3**	**9**	**24**	**Cingulate_Ant_R**
	**13**	**4.93**	**-39**	**-57**	**3**	**Occipital_Mid_L**
		3.89	-36	-69	9	Occipital_Mid_L
	**20**	**4.65**	**-24**	**30**	**48**	**Frontal_Sup_2_L**
	**8**	**4.54**	**-66**	**-27**	**3**	**Temporal_Mid_L**
	**8**	**4.33**	**-39**	**-51**	**24**	**Angular_L**
	**7**	**4.28**	**12**	**45**	**-3**	**Frontal_Med_Orb_R**
	**6**	**4.10**	**18**	**-48**	**39**	**Precuneus_R**
**P1 > HC**	No suprathreshold clusters					

Table S19. Activated regions for the contrast RA (sentence recall) vs. baseline in P1 before surgery. For each peak, the number of voxels in the cluster (k), T value, x, y, z coordinates (in mm) and region label (AAL atlas, Tzourio-Mazoyer et al., 2002) are presented. Activations were obtained at T > 4.59 (*p* < 0.05, FWE). T values marked with * were obtained at T > 3.1 (*p* < 0.001, uncorrected). Regions in bold represent cluster peaks.

Contrast	k	T	x	y	z	AAL
**RA vs. baseline**	**396**	**7.15**	**15**	**-78**	**-18**	**Cerebelum_6_R**
		5.91	27	-63	-24	Cerebelum_6_R
		5.65	6	-84	-6	Lingual_R
	**81**	**6.78**	**-60**	**9**	**0**	**Rolandic_Oper_L**
		5.28	-51	6	12	Frontal_Inf_Oper_L
	**54**	**5.80**	**-39**	**-60**	**-33**	**Cerebelum_Crus1_L**
		4.98	-24	-60	-24	Cerebelum_6_L
	**92**	**5.64**	**-57**	**-15**	**-3**	**Temporal_Mid_L**
		5.44	18	-72	-45	Cerebelum_8_R
	**33**	**5.43**	**-15**	**6**	**72**	**Frontal_Sup_2_L**
	**13**	**4.83**	**0**	**6**	**57**	**Supp_Motor_Area_L**
		4.78	-54	-54	-6	Temporal_Inf_L
	**6**	**4.73**	**-36**	**48**	**18**	**Frontal_Mid_2_L**
	**5**	**4.97**	**-57**	**-24**	**21**	**SupraMarginal_L**
	**39**	**4.88**	**63**	**-12**	**-3**	**Temporal_Sup_R**

**181**	**4.13***	**60**	**3**	**-12**	**Temporal_Pole_Sup_R**
	3.92*	54	-18	-3	Temporal_Sup_R
	4.73*	-36	48	18	Frontal_Mid_2_L
**198**	**4.51***	**-51**	**36**	**12**	**Frontal_Inf_Tri_L**
	4.67*	-33	18	12	Insula_L
	3.58*	-21	18	-3	Putamen_L
**65**	**4.16***	**-24**	**-75**	**-51**	**Cerebelum_7b_L**
	3.93*	-39	-54	-54	Cerebelum_8_L
	4.10*	51	0	51	Frontal_Mid_2_R
**56**	**4.07***	**-18**	**-69**	**57**	**Parietal_Sup_L**
	3.99*	-24	-72	51	Parietal_Sup_L
**14**	**3.70***	**39**	**42**	**30**	**Frontal_Mid_2_R**
**89**	**3.66***	**39**	**48**	**21**	**Frontal_Mid_2_R**
	3.70*	-60	-48	12	Temporal_Sup_L
**39**	**3.66***	**27**	**-39**	**-39**	**Cerebelum_10_R**
	3.59*	39	18	6	Insula_R
**7**	**3.32***	**30**	**9**	**9**	**Putamen_R**

Table S20. Activation differences between P1 and HC during RA (sentence recall) before surgery. For each peak, the number of voxels in the cluster (k), T value, x, y, z coordinates (in mm) and region label (AAL atlas, Tzourio-Mazoyer et al., 2002) are presented. All activations were obtained at T > 3.61 (*p* < 0.001, uncorrected). Regions in bold represent cluster peaks.

Contrast	k	T	x	y	z	AAL
**HC > P1**			No suprathreshold clusters			
**P1 > HC**	**78**	**7.15**	**24**	**-51**	**42**	**Angular_R**
		5.14	27	-51	51	Parietal_Inf_R
		4.83	21	-39	39	Cingulate_Mid_R
	**39**	**5.62**	**-24**	**-60**	**6**	**Calcarine_L**
		4.57	-15	-63	6	Calcarine_L
	**14**	**5.39**	**-57**	**-57**	**-9**	**Temporal_Inf_L**
	**23**	**5.10**	**-12**	**-60**	**-18**	**Cerebelum_6_L**
	**9**	**4.84**	**-39**	**-69**	**-33**	**Cerebelum_Curs1_L**
	**30**	**4.67**	**9**	**-66**	**-18**	**Cerebelum_6_R**
		4.49	0	-66	-15	Vermis_6
	**6**	**4.57**	**36**	**-42**	**-12**	**Fusiform_R**
	**19**	**4.31**	**24**	**-75**	**27**	**Occipital_Sup_R**
	**5**	**4.17**	**9**	**-81**	**-24**	**Cerebelum_Crus1_R**

Table S21. Activated regions for the contrast RA (sentence recall) vs. baseline in P1 after surgery. For each peak, the number of voxels in the cluster (k), T value, x, y, z coordinates (in mm) and region label (AAL atlas, Tzourio-Mazoyer et al., 2002) are presented. Activations were obtained at T > 4.58 (*p* < 0.05, FWE). T values marked with * were obtained at T > 3.1 (*p* < 0.001, uncorrected). Regions in bold represent cluster peaks.

Contrast	k	T	x	y	z	AAL
**RA**	**440**	**9.13**	**-18**	**-96**	**6**	**Occipital_Mid_L**

**vs. baseline**		8.96	-12	-96	18	Occipital_Sup_L
		7.22	-9	-93	-6	Calcarine_L
	**275**	**8.23**	**21**	**-93**	**15**	**Occipital_Sup_R**
		7.52	12	-90	-6	Lingual_R
		5.58	36	-63	-27	Cerebelum_6_R
	**399**	**8.07**	**-42**	**3**	**51**	**Frontal_Mid_2_L**
		7.64	-6	12	72	Supp_Motor_Area_L
		6.44	0	6	54	Supp_Motor_Area_L
	**121**	**7.92**	**-54**	**-30**	**-3**	**Temporal_Mid_L**
	**88**	**6.79**	**12**	**-72**	**6**	**Calcarine_R**
	**43**	**6.06**	**-54**	**6**	**15**	**Precentral_L**
		5.28	-54	6	3	Rolandic_Oper_L
	**29**	**5.54**	**-21**	**-60**	**3**	**Calcarine_L**
	**70**	**5.48**	**-51**	**21**	**18**	**Frontal_Inf_Tri_L**
		5.45	-39	42	15	Frontal_Mid_2_L
	**20**	**5.42**	**-21**	**45**	**27**	**Frontal_Sup_2_L**
	**6**	**5.38**	**-27**	**27**	**-3**	**Insula_L**
	**6**	**5.04**	**-45**	**-42**	**-15**	**Temporal_Inf_L**
	**9**	**4.50***	**54**	**15**	**-18**	**Temporal_Pole_Sup_R**
	**16**	**3.97***	**21**	**-72**	**-51**	**Cerebelum_8_R**
	**10**	**3.95***	**12**	**21**	**30**	**Cingulate_Mid_R**
	**7**	**3.78***	**-12**	**63**	**-3**	**Frontal_Med_Orb_L**
	**14**	**3.68***	**-24**	**9**	**9**	**Putamen_L**
	**5**	**3.33***	**0**	**-72**	**51**	**Precuneus_L**

Table S22. Activation differences between P1 and HC during RA (sentence recall) after surgery. For each peak, the number of voxels in the cluster (k), T value, x, y, z coordinates (in mm) and region label (AAL atlas, Tzourio-Mazoyer et al., 2002) are presented. All activations were obtained at T > 3.61 ($p < 0.001$, uncorrected). Regions in bold represent cluster peaks.

Contrast	k	T	x	y	z	AAL
**HC > P1**	**24**	**6.16**	**-24**	**-33**	**60**	**Postcentral_L**
	**9**	**5.07**	**-48**	**-3**	**-15**	**Temporal_Sup_L**
	**7**	**4.24**	**48**	**-33**	**45**	**SupraMarginal_R**
	**5**	**4.15**	**24**	**-48**	**36**	**Angular_R**
	**6**	**4.06**	**36**	**-27**	**57**	**Precentral_R**
**P1 > HC**	**116**	**7.75**	**-12**	**-93**	**18**	**Occipital_Sup_L**
		5.89	-12	-87	30	Cuneus_L
		5.13	-21	-78	18	Occipital_Mid_L
	**25**	**6.25**	**-24**	**-60**	**6**	**Calcarine_L**
	**34**	**5.07**	**12**	**-72**	**9**	**Calcarine_R**
	**25**	**4.55**	**21**	**-81**	**30**	**Occipital_Sup_R**
	**5**	**4.35**	**15**	**-90**	**18**	**Cuneus_R**
	**17**	**4.33**	**-60**	**-21**	**-12**	**Temporal_Mid_L**
		4.17	-51	-24	-15	Temporal_Mid_L
		3.93	-45	-33	-9	Temporal_Mid_L

	7	4.14	-42	39	15	Frontal_Mid_2_L

Table S23. Activated regions for the contrast GE (generation with implicit encoding) vs. baseline in P2 before surgery. For each peak, the number of voxels in the cluster (k), T value, x, y, z coordinates (in mm) and region label (AAL atlas, Tzourio-Mazoyer et al., 2002) are presented. Activations were obtained at T > 4.64 ($p < 0.05$, FWE). T values marked with * were obtained at T > 3.1 ($p < 0.001$, uncorrected). Regions in bold represent cluster peaks.

Contrast	k	T	x	y	z	AAL
**GE vs. baseline**	**336**	**7.84**	**-3**	**12**	**66**	**Supp_Motor_Area_L**
		7.25	-3	18	42	Frontal_Sup_Medial_L
		6.06	0	15	51	Supp_Motor_Area_L
		6.16	-3	21	36	Cingulate_Mid_L
	**240**	**7.44**	**-51**	**12**	**-3**	**Temporal_Pole_Sup_L**
		6.20	-36	18	-9	Insula_L
		5.86	-48	24	-9	Frontal_Inf_Orb_2_L
		6.41	-51	15	6	Frontal_Inf_Tri_L
	**151**	**6.94**	**12**	**-93**	**9**	**Calcarine_R**
		5.36	12	-99	-9	Lingual_R
		4.44*	33	-75	-15	Fusiform_R
	**92**	**6.81**	**-45**	**6**	**51**	**Frontal_Mid_2_L**
		6.27	-48	6	39	Precentral_L
	**135**	**6.72**	**-36**	**39**	**30**	**Frontal_Mid_2_L**
		5.91	-33	51	15	Frontal_Mid_2_L
		5.20	-21	63	3	Frontal_Sup_2_L
	**34**	**6.51**	**-66**	**-36**	**3**	**Temporal_Mid_L**
		5.18	-63	-42	24	SupraMarginal_L
	**14**	**5.97**	**-66**	**-42**	**21**	**Temporal_Sup_L**
	**85**	**5.75**	**-21**	**-96**	**9**	**Occipital_Mid_L**
		5.57	-27	-93	3	Occipital_Mid_L
		5.47	-42	-84	-9	Occipital_Inf_L
	**14**	**5.51**	**42**	**-81**	**-9**	**Occipital_Inf_R**
	**6**	**5.38**	**39**	**45**	**27**	**Frontal_Mid_2_R**
	**7**	**5.24**	**57**	**-24**	**-3**	**Temporal_Sup_R**
	**6**	**5.14**	**-63**	**-21**	**9**	**Temporal_Sup_L**
	**8**	**5.00**	**-3**	**48**	**42**	**Frontal_Sup_Medial_L**
		4.68	-27	-93	-12	Occipital_Inf_L
	**42**	**4.27***	**24**	**-63**	**63**	**Parietal_Sup_R**
	**60**	**4.25***	**42**	**27**	**-3**	**Frontal_Inf_Orb_2_R**
		3.30*	45	15	-9	Insula_R
		3.21*	54	21	-3	Frontal_Inf_Tri_R
	**6**	**3.58***	**-18**	**-72**	**54**	**Parietal_Sup_L**

Table S24. Activation differences between P2 and HC during GE (generation with implicit encoding) before surgery. For each peak, the number of voxels in the cluster (k), T value, x, y, z coordinates (in mm) and region label (AAL atlas, Tzourio-Mazoyer et al., 2002) are presented. All activations were obtained at T > 3.61 (p < 0.001, uncorrected). Regions in bold represent cluster peaks.

Contrast	k	T	x	y	z	AAL
**HC > P2**	**73**	**10.50**	**3**	**-42**	**12**	**Cingulate_Post_R**
	**36**	**5.80**	**-18**	**6**	**24**	**Caudate_L**
	**5**	**4.70**	**-3**	**-12**	**3**	**Thalamus_L**
	**15**	**4.66**	**-15**	**-87**	**-3**	**Lingual_L**
		3.81	-18	-78	-6	Lingual_L
	**21**	**4.38**	**-9**	**-63**	**-3**	**Lingual_L**
		4.01	-15	-54	-9	Lingual_L
	**6**	**4.25**	**-27**	**-42**	**-27**	**Cerebelum_4_5_L**
	**8**	**4.24**	**9**	**-36**	**48**	**Cingulate_Mid_R**
**P2 > HC**	**19**	**8.87**	**-39**	**-84**	**-6**	**Occipital_Inf_L**
	**13**	**5.67**	**-36**	**18**	**-9**	**Insula_L**
	**6**	**5.36**	**63**	**-3**	**27**	**Postcentral_R**
	**5**	**4.79**	**39**	**45**	**30**	**Frontal_Mid_2_R**
	**7**	**4.78**	**-24**	**60**	**6**	**Frontal_Sup_2_L**
	**13**	**4.25**	**39**	**-69**	**-9**	**Occipital_Inf_R**
	**8**	**4.21**	**-36**	**39**	**27**	**Frontal_Mid_2_L**
	**7**	**4.11**	**3**	**42**	**39**	**Frontal_Sup_Med_L**

Table S25. Activated regions for the contrast GE (generation with implicit encoding) vs. baseline in P2 after surgery. For each peak, the number of voxels in the cluster (k), T value, x, y, z coordinates (in mm) and region label (AAL atlas, Tzourio-Mazoyer et al., 2002) are presented. Activations were obtained at T > 4.65 (p < 0.05, FWE). T values marked with * were obtained at T > 3.1 (p < 0.001, uncorrected). Regions in bold represent cluster peaks.

Contrast	k	T	x	y	z	AAL
**GE vs. baseline**	**128**	**8.64**	**-3**	**9**	**69**	**Supp_Motor_Area_L**
		6.26	0	18	42	Frontal_Sup_Medial_L
		5.28	6	27	33	Cingulater_Mid_R
	**40**	**7.76**	**-12**	**-99**	**15**	**Occipital_Sup_L**
		6.65	-12	-102	6	Occipital_Mid_L
	**128**	**7.23**	**36**	**57**	**12**	**Frontal_Sup_2_R**
		6.38	36	45	30	Frontal_Mid_2_R
		5.41	33	54	24	Frontal_Sup_2_R
	**65**	**7.07**	**-63**	**-36**	**3**	**Temporal_Mid_L**
		6.76	-63	-24	6	Temporal_Sup_L
	**65**	**6.95**	**-48**	**15**	**9**	**Frontal_Inf_Oper_L**
		5.08	-48	24	-6	Frontal_Inf_Orb_2_L
		4.96	-54	18	9	Frontal_Inf_Tri_L
	**84**	**6.58**	**-33**	**33**	**30**	**Frontal_Mid_2_L**
		5.99	-33	45	27	Frontal_Mid_2_L

**36**	**6.56**	**-48**	**3**	**42**	**Precentral_L**
**88**	**6.26**	**0**	**18**	**42**	**Frontal_Sup_Medial_L**
	5.28	6	27	33	Cingualte_Mid_R
**60**	**5.60**	**39**	**27**	**3**	**Insula_R**
	4.49*	57	24	6	Frontal_Inf_Tri_R
	4.55*	54	21	-6	Frontal_Inf_Orb_2_R
	3.57*	42	15	3	Frontal_Inf_Oper_R
**11**	**5.58**	**18**	**-63**	**-54**	**Cerebelum_8_R**
**14**	**5.58**	**-33**	**-90**	**6**	**Occipital_Mid_L**
	5.23	-27	-93	12	Occipital_Mid_L
**9**	**5.34**	**60**	**-18**	**0**	**Temporal_Sup_R**
**22**	**5.27**	**-51**	**27**	**-3**	**Frontal_Inf_Tri_L**
**7**	**5.22**	**48**	**-63**	**-30**	**Cerebelum_Crus1_R**
	4.68	36	-66	-24	Cerebelum_6_R
**20**	**4.99**	**24**	**-96**	**9**	**Occipital_Sup_R**
	4.97	9	-90	9	Calcarine_R
	4.79	15	-99	9	Cuneus_R
**22**	**4.92**	**57**	**6**	**42**	**Precentral_R**
**24**	**4.22***	**66**	**-33**	**9**	**Temporal_Sup_R**
**45**	**4.04***	**-48**	**-78**	**-15**	**Occipital_Inf_L**
**16**	**3.99***	**39**	**-12**	**-39**	**Fusiform_R**

Table S26. Activation differences between P2 and HC during GE (generation with implicit encoding) after surgery. For each peak, the number of voxels in the cluster (k), T value, x, y, z coordinates (in mm) and region label (AAL atlas, Tzourio-Mazoyer et al., 2002) are presented. All activations were obtained at T > 3.61 (p < 0.001, uncorrected). Regions in bold represent cluster peaks.

Contrast	k	T	x	y	z	AAL
**HC > P2**	**49**	**6.60**	**-18**	**-51**	**36**	**Precuneus_L**
		4.66	-9	-57	18	Precuneus_L
	**34**	**5.42**	**-15**	**18**	**-6**	**Putamen_L**
	**15**	**5.40**	**9**	**-42**	**12**	**Cingulate_Post_R**
	**38**	**5.10**	**-24**	**-69**	**15**	**Calcarine_L**
		4.73	-9	-72	18	Calcarine_L
	**20**	**5.01**	**-15**	**-75**	**30**	**Cuneus_L**
	**4**	**4.25**	**54**	**-18**	**-12**	**Temporal_Mid_R**
	**6**	**4.21**	**3**	**-39**	**54**	**Precuneus_R**
	**7**	**4.14**	**-6**	**57**	**3**	**Frontal_Sup_Medial_L**
**P2 > HC**	No suprathreshold clusters					

Table S27. Activated regions for the contrast RECO (recognition) vs. baseline in P2 before surgery. For each peak, the number of voxels in the cluster (k), T value, x, y, z coordinates (in mm) and region label (AAL atlas, Tzourio-Mazoyer et al., 2002) are presented. Activations were obtained at T > 4.64 (p < 0.05, FWE). T values marked with * were obtained at T > 3.1 (p < 0.001, uncorrected). Regions in bold represent cluster peaks.

Contrast	k	T	x	y	z	AAL

**RECO vs. baseline**	**1117**	**9.61**	**36**	**-72**	**-9**	**Occipital_Inf_R**
		9.02	27	-87	9	Occipital_Mid_R
		8.39	27	-60	-15	Fusiform_R
		7.18	45	-57	-9	Temporal_Inf_R
		4.74	45	-54	-3	Temporal_Mid_R
	**901**	**8.94**	**-39**	**-72**	**-12**	**Occipital_Inf_L**
		8.92	-39	-60	-15	Fusiform_L
		8.73	-30	-87	9	Occipital_Mid_L
		6.33	-45	-63	-6	Temporal_Inf_L
		4.69	-45	-54	-3	Temporal_Mid_L
	**83**	**7.51**	**45**	**39**	**3**	**Frontal_Inf_Tri_R**
		5.91	39	42	6	Frontal_Mid_2_R
		4.41	36	27	-6	Frontal_Inf_Orb_2_R
	**194**	**7.32**	**-36**	**-21**	**54**	**Precentral_L**
		5.76	-54	-18	39	Postcentral_L
	**98**	**7.22**	**-27**	**-69**	**30**	**Occipital_Mid_L**
		5.94	-33	-66	51	Parietal_Sup_L
	**37**	**7.00**	**33**	**-63**	**54**	**Parietal_Sup_R**
	**175**	**6.79**	**9**	**21**	**42**	**Frontal_Sup_Medial_R**
		6.45	6	3	60	Supp_Motor_Area_R
		6.31	-6	6	54	Supp_Motor_Area_L
	**119**	**6.65**	**-36**	**15**	**27**	**Frontal_Inf_Tri_L**
		5.21	-36	6	24	Frontal_Inf_Oper_L
	**53**	**6.42**	**39**	**24**	**21**	**Frontal_Mid_2_R**
		5.60	54	3	33	Precentral_R
		4.73	39	9	27	Frontal_Inf_Oper_R
	**34**	**6.20**	**36**	**24**	**-3**	**Insula_R**
	**110**	**5.88**	**-45**	**45**	**18**	**Frontal_Mid_2_L**
		5.85	-39	33	6	Frontal_Inf_Tri_L
	**15**	**5.65**	**-57**	**0**	**39**	**Precentral_L**
	**12**	**5.60**	**54**	**3**	**33**	**Precentral_R**
	**8**	**5.23**	**-51**	**-18**	**21**	**Postcentral_L**
		4.83	-60	-15	21	Postcentral_L
	**9**	**5.01**	**-33**	**21**	**-3**	**Insula_L**
	**24**	**4.76**	**-24**	**33**	**-15**	**OFCpost_L**
		4.27*	-24	33	-12	Frontal_Inf_Orb_2_L
	**33**	**4.32***	**-18**	**-63**	**-54**	**Cerebelum_8_L**
	**13**	**4.01***	**42**	**-18**	**63**	**Precentral_R**
	**6**	**4.01***	**69**	**-33**	**18**	**Temporal_Sup_R**
	**10**	**3.69***	**21**	**42**	**-12**	**OFCant_R**
	**5**	**3.45***	**39**	**39**	**27**	**Frontal_Mid_2_R**
	**5**	**3.44***	**9**	**-75**	**-36**	**Cerebelum_Crus2_R**
	**11**	**3.42***	**-6**	**-78**	**-33**	**Cerebelum_Crus2_L**
	**5**	**3.22***	**18**	**-3**	**-15**	**Hippocampus_R**

Table S28. Activation differences between P2 and HC during RECO (recognition) before surgery. For each peak, the number of voxels in the cluster (k), T value, x, y, z coordinates (in mm) and region label (AAL atlas, Tzourio-Mazoyer et al., 2002) are presented. All activations were obtained at T > 3.61 ($p < 0.001$, uncorrected). Regions in bold represent cluster peaks.

Contrast	k	T	x	y	z	AAL
**HC > P2**	**19**	**4.83**	**-6**	**57**	**6**	**Frontal_Sup_Medial_L**
		3.95	-9	48	6	Frontal_Sup_Medial_L
	**8**	**4.83**	**-18**	**-54**	**6**	**Calcarine_L**
	**46**	**4.74**	**6**	**-60**	**18**	**Calcarine_R**
		4.17	-9	-63	12	Calcarine_L
	**7**	**4.48**	**-21**	**-36**	**-3**	**Hippocampus_L**
	**7**	**4.35**	**-30**	**-21**	**-18**	**Hippocampus_L**
	**5**	**4.29**	**-18**	**33**	**45**	**Frontal_Sup_2_L**
	**6**	**4.12**	**9**	**-60**	**-3**	**Lingual_R**
**P2 > HC**	**41**	**5.71**	**39**	**39**	**3**	**Frontal_Mid_2_R**
	**5**	**4.61**	**-36**	**42**	**24**	**Frontal_Mid_2_L**
	**5**	**4.50**	**-9**	**12**	**-6**	**Caudate_L**
	**7**	**4.29**	**-33**	**33**	**9**	**Frontal_Inf_Tri_L**
	**5**	**4.14**	**-45**	**42**	**12**	**Frontal_Inf_Tri_L**

Table S29. Activated regions for the contrast RECO (recognition) vs. baseline in P2 after surgery. For each peak, the number of voxels in the cluster (k), T value, x, y, z coordinates (in mm) and region label (AAL atlas, Tzourio-Mazoyer et al., 2002) are presented. Activations were obtained at T > 4.65 ($p < 0.05$, FWE). T values marked with * were obtained at T > 3.1 ($p < 0.001$, uncorrected). Regions in bold represent cluster peaks.

Contrast	k	T	x	y	z	AAL
**RECO vs. baseline**	**1263**	**9.73**	**39**	**-69**	**-12**	**Occipital_Inf_R**
		9.60	21	-84	-12	Lingual_R
		9.36	36	-90	-9	Occipital_Inf_R
		8.43	42	-63	-9	Temporal_Inf_R
		3.83	36	-27	-6	Hippocampus_R
	**960**	**9.48**	**-36**	**-69**	**-15**	**Fusiform_L**
		7.49	-51	-66	-12	Occipital_Inf_L
		7.44	-39	-81	-6	Occipital_Inf_L
	**454**	**7.75**	**-42**	**-15**	**54**	**Precentral_L**
		6.89	-54	-15	39	Postcentral_L
		4.91	-60	-21	9	Temporal_Sup_L
		3.64*	-60	-24	15	SupraMarginal_L
	**62**	**7.20**	**45**	**42**	**3**	**Front_Mid_2_R**
		4.02*	36	51	9	Frontal_Sup_2_R
	**99**	**6.08**	**18**	**-63**	**-54**	**Cerebelum_8_R**
		5.29	0	-66	-36	Vermis_8
	**40**	**5.82**	**0**	**0**	**66**	**Supp_Motor_Area_L**
		4.88	-9	-9	72	Supp_Motor_Area_L
	**15**	**5.76**	**-63**	**3**	**27**	**Precentral_L**

**48**	**5.66**	**57**	**0**	**39**	**Precentral_R**
	5.56	60	-12	39	Postcentral_R
	4.22*	63	-30	18	Temporal_Sup_R
**18**	**5.54**	**60**	**-15**	**21**	**Postcentral_R**
	5.43	63	-24	21	SupraMarginal_R
**16**	**5.50**	**-39**	**33**	**9**	**Front_Inf_Tri_L**
**50**	**5.38**	**0**	**0**	**51**	**Supp_Motor_Area_L**
	5.16	3	18	42	Frontal_Sup_Medial_L
**9**	**5.22**	**-6**	**-9**	**54**	**Supp_Motor_Area_L**
**85**	**4.79**	**36**	**-30**	**39**	**Postcentral_R**
	3.54*	30	-45	42	Angular_R
	3.28*	42	-42	48	Parietal_Inf_R
**57**	**4.70**	**24**	**36**	**-15**	**OFCant_R**
**58**	**4.53***	**39**	**24**	**21**	**Frontal_Mid_2_R**
**24**	**4.35***	**18**	**66**	**3**	**Frontal_Sup_2_R**
**61**	**4.24***	**-6**	**-24**	**9**	**Thalamus_L**
**46**	**4.19***	**30**	**15**	**63**	**Frontal_Sup_2_R**
**36**	**4.02***	**-33**	**21**	**-9**	**Insula_L**
**35**	**3.99***	**-30**	**-69**	**48**	**Parietal_Sup_2_L**
**29**	**3.99***	**33**	**-66**	**54**	**Parietal_Sup_R**
**13**	**3.97***	**-24**	**33**	**-15**	**OFCpost_L**
**23**	**3.93***	**57**	**21**	**33**	**Frontal_Inf_Oper_R**
**8**	**3.93***	**45**	**-3**	**-33**	**Temporal_Inf_R**
**12**	**3.92***	**3**	**54**	**-9**	**Frontal_Med_Orb_R**
**13**	**3.71***	**-15**	**51**	**-9**	**Frontal_Sup_2_L**
**50**	**3.65***	**15**	**-18**	**15**	**Thalamus_R**
	3.53*	24	-18	24	Caudate_R
**5**	**3.64***	**-18**	**63**	**6**	**Frontal_Sup_2_L**
**6**	**3.60***	**45**	**-33**	**21**	**Rolandic_Oper_R**
**9**	**3.54***	**-6**	**42**	**-9**	**Frontal_Med_Orb_L**
**5**	**3.50***	**-18**	**-3**	**18**	**Caudate_L**
**8**	**3.48***	**-36**	**-60**	**36**	**Angular_L**
**6**	**3.42***	**0**	**-36**	**27**	**Cingulate_Post_L**

Table S30. Activation differences between P2 and HC during RECO (recognition) after surgery. For each peak, the number of voxels in the cluster (k), T value, x, y, z coordinates (in mm) and region label (AAL atlas, Tzourio-Mazoyer et al., 2002) are presented. All activations were obtained at T > 3.61 (p < 0.001, uncorrected). Regions in bold represent cluster peaks.

**Contrast**	**k**	**T**	**x**	**y**	**z**	**AAL**
**HC > P2**	No suprathreshold clusters					
**P2 > HC**	**17**	**5.65**	**-60**	**-6**	**33**	**Precentral_L**
	**5**	**4.70**	**-6**	**33**	**48**	**Frontal_Sup_Medial_L**
	**5**	**4.34**	**-9**	**-30**	**9**	**Thalamus_L**
	**8**	**4.10**	**42**	**42**	**3**	**Frontal_Mid_2_R**

Table S31. Activated regions for the contrast RA (sentence recall) vs. baseline in P2 before surgery. For each peak, the number of voxels in the cluster (k), T value, x, y, z coordinates (in mm) and region label (AAL atlas, Tzourio-Mazoyer et al., 2002) are presented. Activations were obtained at T > 4.64 ($p < 0.05$, FWE). T values marked with * were obtained at T > 3.1 ($p < 0.001$, uncorrected). Regions in bold represent cluster peaks.

Contrast	k	T	x	y	z	AAL
**RA vs. baseline**	**664**	**15.11**	**-12**	**-102**	**6**	**Occipital_Mid_L**
		11.48	-30	-90	9	Occipital_Mid_L
		11.26	-24	-96	-12	Occipital_Inf_L
	**525**	**11.36**	**15**	**-96**	**-6**	**Calcarine_R**
		11.32	24	-87	-9	Lingual_R
	**204**	**9.10**	**-63**	**-21**	**9**	**Temporal_Sup_L**
		7.31	-66	-39	6	Temporal_Mid_L
	**168**	**7.86**	**60**	**-21**	**0**	**Temporal_Sup_R**
		6.40	63	-33	3	Temporal_Sup_R
		6.31	63	-6	-9	Temporal_Sup_R
	**88**	**7.66**	**30**	**-66**	**60**	**Parietal_Sup_R**
	**60**	**6.93**	**-36**	**21**	**-9**	**Frontal_Inf_Orb_2_L**
	**91**	**6.60**	**3**	**6**	**63**	**Supp_Motor_Area_R**
		5.63	-12	9	69	Supp_Motor_Area_L
	**90**	**6.58**	**-48**	**3**	**48**	**Precentral_L**
		5.42	-51	12	30	Precentral_L
		5.40	-36	3	60	Front_Mid_2_L
	**10**	**5.90**	**-18**	**63**	**3**	**Front_Sup_2_L**
	**119**	**5.89**	**-39**	**45**	**6**	**Frontal_Inf_Tri_L**
		5.15	-39	42	24	Frontal_Mid_2_L
		5.31	-51	12	6	Frontal_Inf_Oper_L
	**20**	**5.72**	**-48**	**15**	**6**	**Frontal_Inf_Tri_L**
	**18**	**5.34**	**-18**	**-72**	**57**	**Parietal_Sup_L**
		3.90	-39	-57	42	Angular_L
	**15**	**5.23**	**6**	**21**	**42**	**Frontal_Sup_Medial_R**
		4.91	-3	18	42	Frontal_Sup_Medial_L
	**7**	**5.21**	**-30**	**9**	**66**	**Frontal_Mid_2_L**
	**22**	**5.17**	**-33**	**30**	**18**	**Frontal_Inf_Tri_L**
		5.12	-33	21	21	Frontal_Inf_Tri_L
	**7**	**5.16**	**36**	**24**	**0**	**Insula_R**
	**55**	**4.97**	**57**	**3**	**42**	**Precentral_R**
	**29**	**4.78**	**39**	**24**	**21**	**Frontal_Mid_2_R**
	**37**	**4.58**	**-3**	**48**	**48**	**Frontal_Sup_Medial_L**
	**32**	**4.11***	**42**	**-39**	**54**	**Parietal_Inf_R**
	**30**	**4.06***	**45**	**-66**	**-33**	**Cerebelum_Crus1_R**
	**8**	**4.02***	**39**	**-15**	**-39**	**Fusiform_R**
	**27**	**3.92***	**63**	**-24**	**45**	**SupraMarginal_R**
	**27**	**3.85***	**-12**	**-57**	**72**	**Precuneus_L**
	**11**	**3.83***	**-21**	**30**	**-21**	**OFCant_L**
	**16**	**3.73***	**-9**	**48**	**-9**	**Frontal_Med_Orb_L**

**24**	**3.71***	**9**	**12**	**3**	**Caudate_R**
**10**	**3.67***	**27**	**-60**	**33**	**Occipital_Sup_R**
**6**	**3.56***	**45**	**-36**	**9**	**Temporal_Sup_R**
**7**	**3.43***	**-15**	**-3**	**18**	**Caudate_L**
**12**	**3.38***	**18**	**-48**	**-24**	**Cerebelum_4_5_R**

Table S32. Activation differences between P2 and HC during RA (sentence recall) before surgery. For each peak, the number of voxels in the cluster (k), T value, x, y, z coordinates (in mm) and region label (AAL atlas, Tzourio-Mazoyer et al., 2002) are presented. All activations were obtained at T > 3.61 (p < 0.001, uncorrected). Regions in bold represent cluster peaks.

Contrast	k	T	x	y	z	AAL
**HC > P2**	**194**	**6.39**	**-30**	**-63**	**-9**	**Fusiform_L**
		5.85	-18	-51	-9	Lingual_L
		5.51	6	-60	-3	Lingual_R
	**101**	**6.20**	**15**	**-72**	**21**	**Cuneus_R**
		5.19	12	-75	9	Calcarine_R
	**62**	**5.70**	**-15**	**-84**	**-3**	**Lingual_L**
		4.68	-6	-84	15	Cuneus_L
		4.55	-15	-81	18	Occipital_Sup_L
	**10**	**4.42**	**-12**	**-69**	**15**	**Calcarine_L**
	**5**	**4.17**	**-24**	**-69**	**15**	**Calcarine_L**
	**8**	**3.98**	**24**	**-51**	**6**	**Calcarine_R**
**P2 > HC**	**13**	**5.56**	**-39**	**-84**	**-9**	**Occipital_Inf_L**
		4.67	-33	-90	0	Occipital_Mid_L
	**14**	**5.37**	**27**	**-63**	**60**	**Parietal_Sup_R**
	**16**	**5.21**	**-36**	**-72**	**-18**	**Fusiform_L**
	**7**	**4.39**	**-12**	**-96**	**21**	**Cuneus_L**
	**23**	**4.16**	**21**	**-96**	**-6**	**Cacalrine_R**
		4.13	21	-87	-12	Lingual_R

Table S33. Activated regions for the contrast RA (sentence recall) vs. baseline in P2 after surgery. For each peak, the number of voxels in the cluster (k), T value, x, y, z coordinates (in mm) and region label (AAL atlas, Tzourio-Mazoyer et al., 2002) are presented. Activations were obtained at T > 4.65 (p < 0.05, FWE). T values marked with * were obtained at T > 3.1 (p < 0.001, uncorrected). Regions in bold represent cluster peaks.

Contrast	k	T	x	y	z	AAL
**RA vs. baseline**	**92**	**7.37**	**60**	**-18**	**-3**	**Temporal_Sup_R**
		6.4	66	-12	6	Temporal_Sup_R
		3.7*	60	-18	12	Rolandic_Oper_R
		3.5*	60	-27	-3	Temporal_Mid_R
		3.28*	57	6	-9	Temporal_Pole_Sup_R
	**37**	**6.88**	**-60**	**-18**	**6**	**Temporal_Sup_L**
		4.79	-57	-18	0	Temporal_Mid_L
	**6**	**3.52***	**57**	**0**	**45**	**Precentral_L**

Table S34. Activation differences between P2 and HC during RA (sentence recall) after surgery. For each peak, the number of voxels in the cluster (k), T value, x, y, z coordinates (in mm) and region label (AAL atlas, Tzourio-Mazoyer et al., 2002) are presented. All activations were obtained at T > 3.61 ($p < 0.001$, uncorrected). Regions in bold represent cluster peaks.

Contrast	k	T	x	y	z	AAL
**HC > P2**	**54**	**6.56**	**51**	**18**	**36**	**Frontal_Inf_Oper_R**
		3.95	39	18	24	Frontal_Inf_Tri_R
	**76**	**6.56**	**-39**	**-51**	**48**	**Parietal_Inf_L**
		5.68	-45	-39	51	Parietal_Inf_L
	**72**	**6.22**	**-48**	**9**	**24**	**Frontal_Inf_Oper_L**
	**46**	**5.57**	**-45**	**39**	**12**	**Frontal_Inf_Tri_L**
	**25**	**5.39**	**42**	**-63**	**51**	**Angular_R**
		3.87	39	-57	42	Parietal_Inf_R
	**58**	**5.35**	**-9**	**33**	**45**	**Frontal_Sup_medial_L**
		5.06	9	33	48	Frontal_Sup_medial_R
	**51**	**5.25**	**9**	**-75**	**-27**	**Cerebelum_Crus1_R**
		5.11	9	-78	-36	Cerebelum_Crus2_R
		4.33	-3	-75	-30	Cerebelum_Crus2_L
	**9**	**4.47**	**51**	**39**	**15**	**Frontal_Inf_Tri_R**
	**7**	**4.40**	**30**	**-78**	**45**	**Occipital_Sup_R**
	**11**	**4.39**	**-24**	**-69**	**48**	**Patietal_Sup_L**
	**8**	**4.34**	**-15**	**-87**	**-3**	**Lingual_L**
**P2 > HC**	No suprathreshold clusters					
